# Design, Modeling and Performance Analysis of an Actively Variable Stiffness Pneumatic Flexible Bending Joint

**DOI:** 10.3390/s26165200

**Published:** 2026-08-17

**Authors:** Xia Wang, Haoran Yuan, Pei Wang, Peng Gao, Honghao Xing, Mingyang Han, He Peng

**Affiliations:** 1 College of Mechanical Engineering, Beihua University, Jilin 132021, China; wangxiabh@163.com (X.W.); 18738803565@163.com (H.Y.); pei_0506@163.com (P.W.); 18333636588@163.com (H.X.); 17386807583@163.com (M.H.); 2 Beijing Spacecraft Manufacturing Co., Ltd., Beijing 100094, China; gao-peng1010@163.com

**Keywords:** actively variable stiffness, pneumatic actuation, flexible bending joint, performance analysis, particle jamming

## Abstract

**Highlights:**

**What are the main findings?**

- A positive-pressure double-airbag gap-constrained particle-jamming method is proposed, and a novel actively variable-stiffness pneumatic flexible bending joint is developed.
- Theoretical models of the joint bending angle and tangential stiffness are established and experimentally validated.

**What are the implications of the main findings?**

- The positive-pressure double-airbag gap-constrained particle-jamming method maintains a relatively uniform particle distribution during joint deformation, while the variable stiffness device enables active continuous stiffness modulation of the joint.
- The validated models quantitatively describe the bending response to actuation pressure and the variation in tangential stiffness with bending angle.

**Abstract:**

The contradiction between high compliance and low load-bearing capacity of flexible manipulators limits their engineering applications. Meanwhile, the theoretical modeling of the deformation and variable stiffness characteristics of flexible joints still faces considerable challenges. This paper proposes a positive-pressure double-airbag gap-constrained particle-jamming variable stiffness method and develops a novel actively variable stiffness pneumatic flexible bending joint with an integrated configuration of actuator, variable stiffness device (VSD), and primary structure. Based on classical elasticity theory and Coulomb–Amontons’ law of friction, theoretical models for the bending angle and tangential stiffness are established and verified through prototype experiments. With VSD activation, the joint reaches a bending angle of 56.35° at 0.4 MPa. At 40° forward bending, VSD activation increases the tangential stiffness from 0.167 N/mm to 0.832 N/mm, with the stiffness ratio between 40° and 0° increasing from 1.56 without VSD to 4.80 with VSD activation. Model predictions agree well with experimental data, yielding mean relative errors of 6.77% for the bending-angle model with VSD and 6.76% for the forward tangential-stiffness model with VSD activation. A coupling effect between bending deformation and stiffness is observed. The results demonstrate that the proposed joint achieves substantial stiffness regulation, providing a basis for its application in flexible robotic systems.

## 1. Introduction

Technological progress and population aging have jointly driven the continuous growth in the demand for service robots in fields such as medical rehabilitation, home services, and commercial catering [1,2,3]. Traditional rigid robots are mostly made of high-modulus materials and offer high efficiency and precision. However, their structural characteristics limit their application in unstructured environments and compromise human–robot interaction safety [4,5]. Flexible robots have attracted increasing research attention in the field of service robots because of their high flexibility and favorable human–robot interaction performance. Recent advances in integrated actuation and sensing have further promoted the development of intelligent soft robots [6]. Meanwhile, the development of wearable robotic systems for rehabilitation and assistance has also increased the demand for compact and lightweight robotic structures [7]. However, the contradiction between the flexibility of flexible bending joints and the load-bearing capacity has become a key scientific problem restricting their practical application [8].

To improve the stiffness of flexible robots, researchers have proposed variable stiffness technology, which introduces variable stiffness structures into the design of flexible robots to enhance stiffness and load-bearing capacity, thereby expanding their application scope [9,10,11,12]. At present, the main variable stiffness methods include jamming-based variable stiffness, structure-interference-based variable stiffness, and smart material-based variable stiffness [13,14,15,16].

The particle jamming variable stiffness technology adjusts the stiffness of flexible joints by changing the state of particles. To improve the adaptability of flexible robots to unstructured environments, the particles are in a loose, flowable state in the initial stage. Through external interference, the particles aggregate and solidify, and the variable stiffness function is realized by adjusting the degree of particle solidification [17,18]. The driving methods for particle jamming variable stiffness mainly include negative-pressure, positive-pressure, piston-driven, and magnetically driven types [19].

A typical example is the universal flexible gripper proposed by Amend et al. based on negative-pressure particle jamming, which enables adaptive grasping of irregular objects [20]. Liu et al. proposed a positive-pressure particle-jamming (PPJ) variable-stiffness structure to alleviate the pressure-range limitation of conventional negative-pressure particle jamming. Stiffness regulation is achieved by using an inflatable inner chamber to compress the outer particle-jamming layer, thereby extending the available operating-pressure range beyond that of negative-pressure systems [21].

Peng et al. proposed a dual-chamber particle-jamming soft robotic gripper with an independently decoupled inner actuation chamber and outer particle-jamming layer. Continuous stiffness regulation is achieved by adjusting the vacuum pressure while maintaining compliant wrapping during large deformation [22]. Wang et al. proposed a jammed adjustable pneumatic-network soft actuator that combines magnetically controlled layer jamming with particle jamming. The actuator enables programmable bending and multidirectional force regulation while maintaining continuous deformation [23].

Structural-interference-based variable stiffness technology improves the stiffness and load-bearing capacity of flexible robots through structural design and external interference [24]. Inspired by the structural characteristics of scaly organisms such as pangolins and fish, Wang et al. and Chen et al. proposed a programmable robot with a stratified scale structure (SAILS). Actuation is realized through a reversely designed programmable scale array, which collaboratively achieves shape deformation and stiffness regulation within a highly integrated and compact structure [25]. Wang et al. developed a humanoid forearm-inspired rotary manipulator based on Kresling pneumatic origami actuators and a rigid–flexible hybrid structure. The antagonistic actuator arrangement enables large-angle rotation and high-torque output [26].

Smart-material-based methods achieve reversible switching between soft and rigid states through thermal, electrical, magnetic, or pressure stimuli [27]. These stimuli change the molecular-chain state, interfacial friction, or particle-packing state of the material. Copaci et al. developed a flexible shape-memory-alloy actuator for soft robotic and wearable applications. The actuator combines the high force-to-weight ratio of Ni–Ti alloy with a Bowden transmission to achieve large-displacement actuation and has been applied to flexible exoskeleton systems [28].

In summary, existing variable stiffness technologies provide different approaches to balancing compliance and load capacity, although each method has specific advantages and limitations. The negative-pressure particle-jamming method has a fast response speed but a narrow variable stiffness range limited by 1 atm. The positive-pressure particle-jamming method achieves a large variable stiffness range, yet it is prone to non-uniform particle distribution. The piston-based particle-jamming method is less suitable for continuous stiffness regulation. The magnetically driven method delivers fast response but is vulnerable to external environmental interference [29]. The structural-interference method offers high motion efficiency, although its fixed structure limits its range of applications [30,31]. The smart-material method has a large variable stiffness range, but it features slow response speed and high environmental dependence [32].

Against this background, this study proposes a positive-pressure double-airbag gap-constrained particle-jamming method that addresses three specific limitations of existing approaches. First, positive-pressure actuation up to 0.4 MPa extends the available pressure range. Second, the double-airbag gap-constrained configuration mitigates excessive local particle accumulation during joint deformation. Third, pneumatic regulation of the VSD provides active and continuous stiffness adjustment. The detailed particle-confinement and jamming mechanisms are described in Section 2.1. Meanwhile, a novel actively variable stiffness pneumatic flexible bending joint is developed using an integrated configuration of the actuator, VSD, and primary structure. The joint combines a compact layout with enhanced stiffness and pose-retention capability while maintaining flexibility and compliance, providing a potential design basis for flexible robotic systems.

## 2. Principle and Design

### 2.1. Working Principle of the Pneumatic Flexible Bending Joint

**Figure 1:**
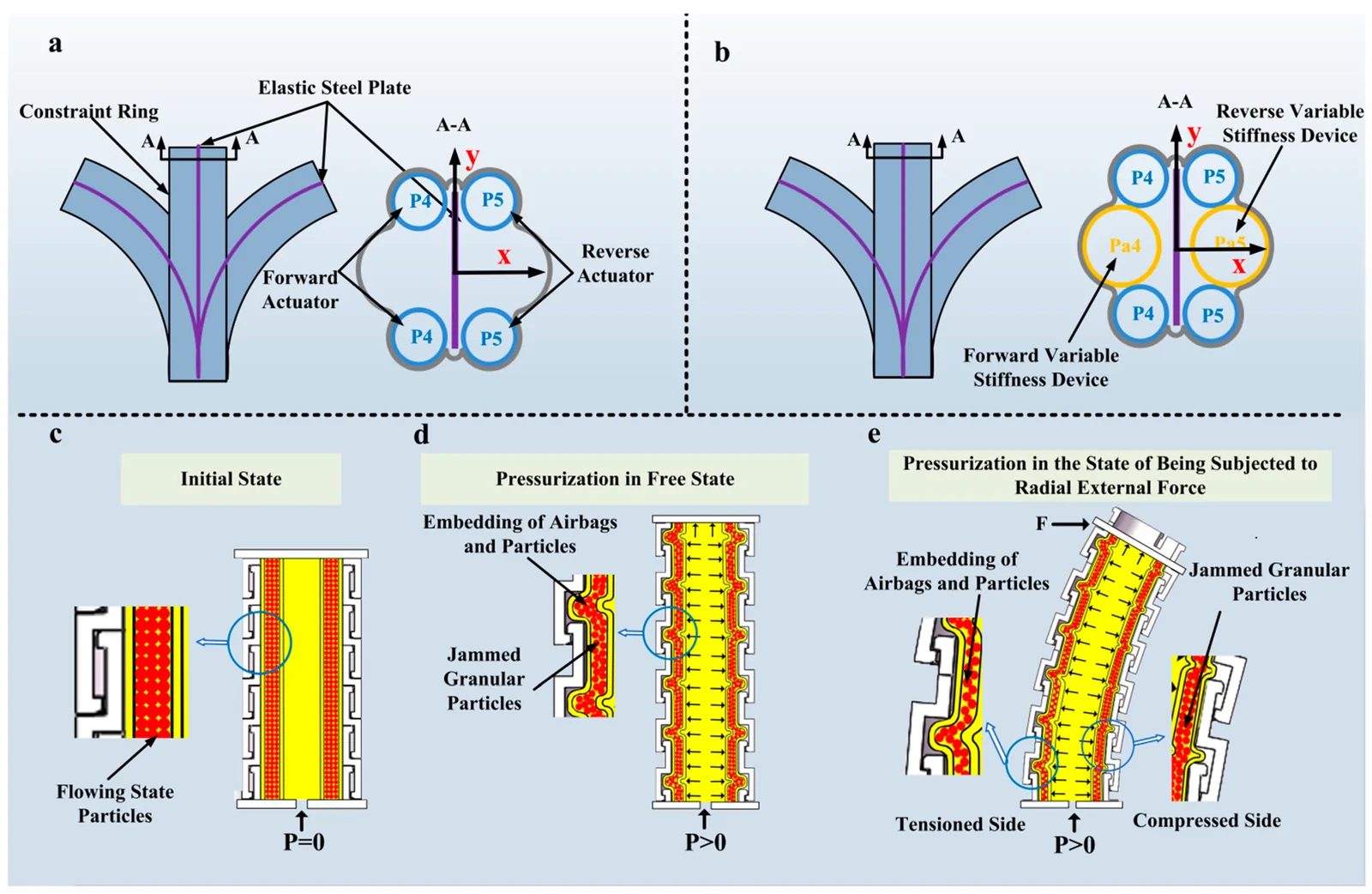
Working principle of flexible bending joint and variable stiffness device: (**a**) working principle of pneumatic flexible bending unit; (**b**) working principle of the actively variable stiffness pneumatic flexible bending joint; (**c**) initial state of variable stiffness adjustment unit; (**d**) pressurization of variable stiffness adjustment unit in free state; (**e**) pressurization of variable stiffness adjustment unit under radial external force.

The self-developed artificial muscle serves as both the actuator and structural body of the joint, thereby maintaining structural and actuation flexibility while enabling a compact and lightweight design. The forward and reverse actuators are symmetrically arranged on both sides of the elastic steel plate. The constraint rings restrict the radial expansion of the artificial muscles. By adjusting the pressure difference between the two actuator groups, the joint achieves one-dimensional bidirectional bending. When the forward actuator is pressurized (P4 > 0), the artificial muscle undergoes axial elongation under the radial constraint of the constraint rings. Because the axial stiffness of the central elastic steel plate is much greater than that of the flexible base, the elongation is constrained by the steel plate. With the elastic steel plate serving as the neutral layer, the flexible bending unit therefore bends toward the unpressurized reverse artificial muscle, producing motion along the positive *x*-axis (referred to as forward bending). Similarly, when the reverse artificial muscle is filled with air pressure (P5 > 0), the flexible bending unit will bend in the opposite direction of the *x*-axis (referred to as reverse bending) (Figure 1a).

To enhance the joint’s load-bearing capacity, a variable stiffness device (VSD) is introduced and placed on both sides of the elastic steel plate, symmetrically installed between the two artificial muscles. The pressure value of the VSD is adjusted to achieve real-time online adjustment of the joint stiffness (Figure 1b). Based on the functional and structural characteristics of the VSD, the actively variable stiffness pneumatic flexible bending joint can adopt two variable stiffness methods: (1) In the drive-based variable stiffness method, the VSD simultaneously performs the functions of stiffness adjustment and driving. The forward (or reverse) VSD and the forward (or reverse) artificial muscle are simultaneously pressurized (Pa4 = P4 > 0 or Pa5 = P5 > 0), and the flexible bending joint bends in the forward (or reverse) direction. During the joint movement, the stiffness changes in real time. (2) In the antagonistic variable stiffness method, the forward actuator (P4 > 0) or the reverse artificial muscle (P5 > 0) is pressurized first, driving the flexible bending joint to the target position. Then, the forward and reverse VSD are simultaneously pressurized (Pa4 = Pa5), and the joint stiffness is adjusted through the antagonistic action of the VSD.

The variable stiffness device adopts positive-pressure actuation, a double-airbag particle-filled cavity structure, and a gap constraint structure. Positive-pressure actuation effectively expands the variable stiffness range. The particles are confined within the cavity between the inner and outer air chambers. During joint bending, both air chambers deform simultaneously, allowing the particles to remain relatively uniformly distributed between them before and after deformation. Pressure regulation of the inner air chamber enables active continuous stiffness modulation. The gap constraint structure can further improve the variable stiffness capability through particle embedding. In the initial state (P = 0), particles are in a free-flowing state, and the VSD presents low stiffness characteristics (Figure 1c). When the inner air chamber is pressurized (P > 0), radial expansion occurs first, squeezing particles and driving the outer air chamber to embed into the slot of the gap constraint ring. The particles are rapidly compacted in the confined space and form frictional locking with the slot wall, constructing a stable slot-blocking structure, which significantly increases the stiffness of the VSD (Figure 1d). At this time, the joint bends under external force. The reduction in the gap on the compressed side further strengthens the slot blocking effect of particles, while the blocking effect on the tension side is weakened (Figure 1e). After the inner air chamber is depressurized, the squeezing force on the particles disappears. The embedded particles disengage from the slot under the restoring force of the outer air chamber, regain the ability of free sliding and rearrangement, and the stiffness of the VSD returns to the initial low-stiffness state.

### 2.2. Design and Development of Flexible Bending Joint

**Figure 2:**
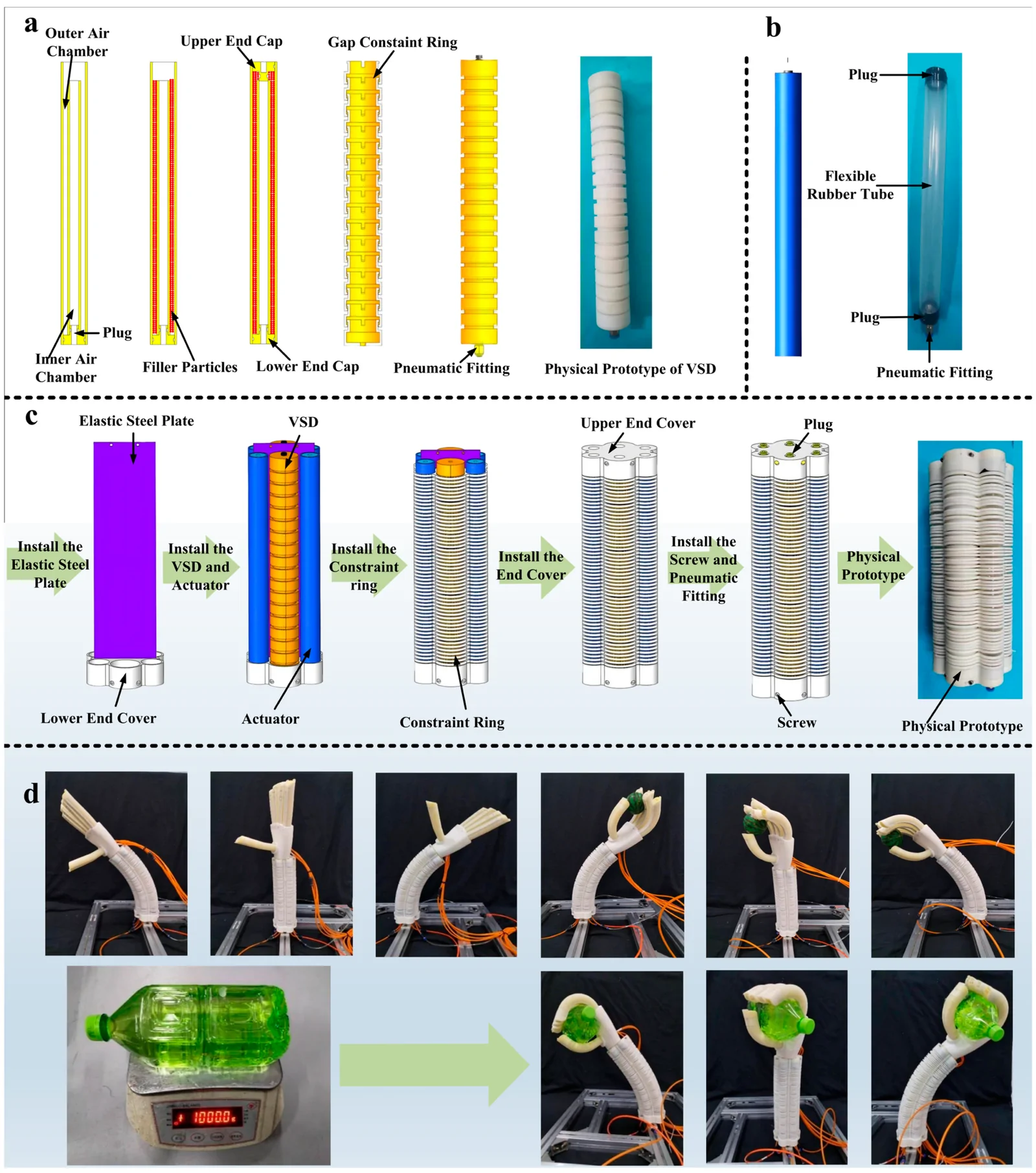
Structural design, development, and functional validation of the flexible bending joint: (**a**) manufacturing and prototype of the variable stiffness device; (**b**) actuation unit and its prototype; (**c**) development and prototyping of the actively variable stiffness pneumatic flexible bending joint; (**d**) grasping tests of the flexible bending joint under different pressure conditions, demonstrating stable grasping capability for a 1.0 kg bottle.

The variable stiffness device adopts a coaxial multi-layer nested structure. From the inner to the outer layers, it consists of an inner air chamber, particles, an outer air chamber, and gap constraint rings (Figure 2a). Both the inner and outer air chambers are fabricated from commercially available silicone–fluororubber tubes, which effectively reduces manufacturing cost. The intermediate cavity between the two chambers is filled with granular particles and is sealed by stepped upper and lower metal plugs, while the dual-chamber configuration further enhances the stability of particle distribution. A set of gap constraint rings is axially mounted on the outer side of the outer air chamber. These constraint rings adopt an interlocking nested structure that accommodates compression, extension, and bending deformations. During bending, they provide sufficient external constraint to the outer air chamber, thereby preventing constraint instability induced by pressurization. Upper and lower end caps are fixed at both ends of the device. The lower end cap is designed with a threaded port for installing a pneumatic fitting, through which pressurized gas is supplied to achieve variable stiffness functionality. The actuation unit employs a self-developed extensible pneumatic artificial muscle (Figure 2b). A sealing connector is installed at the upper end of the actuator to ensure the airtightness of the internal cavity, while a threaded port is provided at the lower end for mounting a pneumatic fitting, enabling the input of pressurized gas to realize actuation.

The actively variable stiffness pneumatic flexible bending joint primarily consists of an elastic steel plate, a variable stiffness device, a pneumatic artificial muscle, constraint rings, and upper and lower end caps (Figure 2c). The elastic steel plate is mounted at the neutral layer of the lower end cap of the joint, providing enhanced torsional and tensile strength. The actuator and the variable stiffness device are symmetrically arranged on both sides of the elastic steel plate at corresponding positions. Constraint rings are installed along the axial direction in a modular configuration to restrict the radial deformation of the pneumatic artificial muscle. Protrusions are designed on both the upper and lower surfaces of each constraint ring to ensure uniform axial spacing after assembly, thereby increasing the achievable bending angle of the joint. Finally, the upper end cap is installed to complete the assembly of the actively variable stiffness pneumatic flexible bending joint. Grasping tests of the flexible bending joint under different pressure conditions are shown (Figure 2d). The key parameters of the joint are listed in Table 1.

## 3. Static Modeling and Analysis of the Actively Variable Stiffness Pneumatic Flexible Bending Joint

### 3.1. Theoretical Model of Bending Angle

To characterize the bending behavior and stiffness modulation characteristics of the actively variable stiffness pneumatic flexible bending joint, theoretical models for the bending angle and tangential stiffness are established, taking forward bending as a representative case, thereby providing a theoretical basis for motion control and performance optimization.

Modeling assumptions. To simplify the theoretical derivation, the following assumptions and simplifications are adopted:

(1) To simplify the theoretical analysis, the artificial muscle is assumed to undergo uniform axial deformation during joint bending.
(2) The artificial muscles and VSD air chambers are made of isotropic and incompressible rubber; therefore, the volume of their rubber walls remains constant during deformation.
(3) The elastic steel plate is treated as a slender beam whose deformation is dominated by bending. Therefore, its transverse shear deformation is neglected in the theoretical model.
(4) The friction generated by particle squeezing on the actively pressurized side is retained because it is a primary source of stiffness enhancement. When the unpressurized VSD is passively compressed, its elastomeric rubber air chambers undergo passive deformation, and the resulting internal friction is relatively small and therefore neglected. However, the deformation resistance moments of both the inner and outer air chambers are retained.

### 3.2. Bending Angle Model

**Figure 3:**
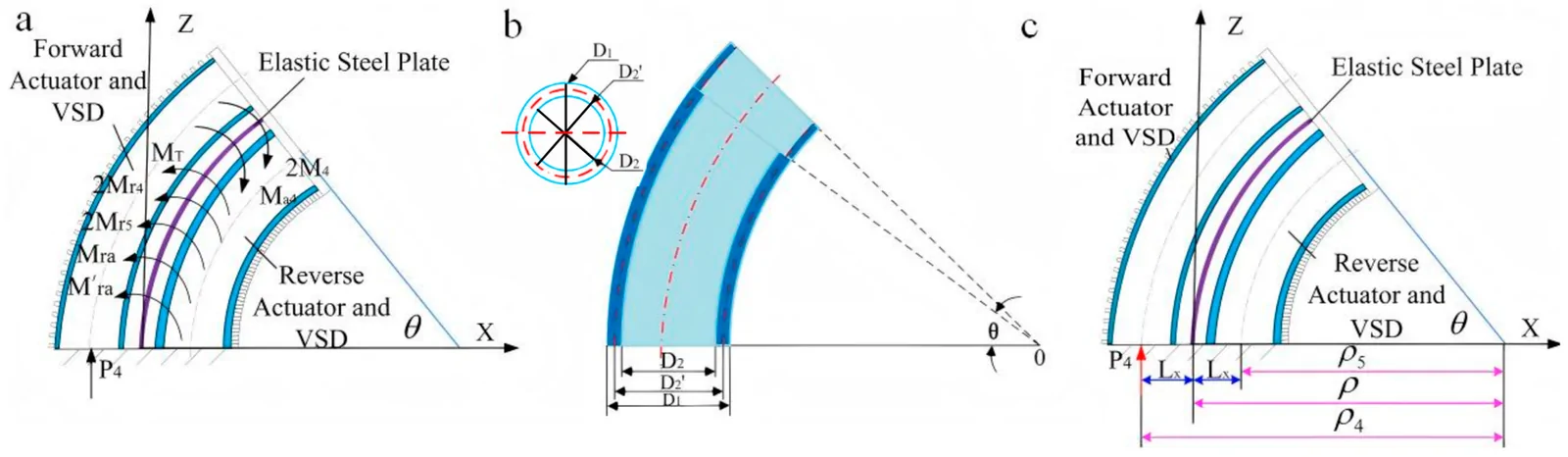
(**a**) Bending moment analysis of the VSD flexible bending joint; (**b**) deformation analysis of the artificial muscle; (**c**) geometric relationship of VSD flexible bending joint deformation.

The forward actuator and forward VSD are pressurized simultaneously, causing the VSD flexible bending joint to bend forward. The bending moment analysis of the VSD flexible bending joint is presented in Figure 3a.

The moment equilibrium equation of the active VSD flexible bending joint is given by

(1) $$2M_{4}+M_{a4}=M_{T}+2M_{r4}+2M_{r5}+M_{ra}+M\prime_{ra}$$

where $M_{4}$ and $M_{a4}$ are the driving moments generated by the pressurized forward actuator and forward VSD, respectively; $M_{T}$ is the resisting moment of the elastic steel plate; $M_{r4}$ and $M_{r5}$ are the resisting moments of the forward and reverse actuators, respectively; and $M_{ra}$ and $M\prime_{ra}$ are the resisting moments of the forward and reverse VSDs, respectively.

When the forward actuator is pressurized, the generated output torque is given by

(2) $$M_{4}=P_{4}S\prime_{4}L_{x}$$

where $P_{4}$ is the pressure applied to the forward actuator, $S\prime_{4}$ is the cross-sectional area of the forward artificial-muscle cavity after deformation, and $L_{x}$ is the distance from the central axis of the forward artificial muscle to the centerline of the elastic steel plate.

When the forward actuator is pressurized, the artificial muscle undergoes elongation deformation, and the cross-sectional area of the elastic rubber tube changes. Its outer diameter remains constrained and unchanged, whereas its inner diameter increases and the thickness of the elastic rubber tube decreases (Figure 3b). Therefore, the axial deformation of the artificial muscle is mainly manifested as a geometric nonlinear problem. The volume of a single artificial muscle before the deformation of the forward actuator is given by

(3) $$V_{4}=\frac{\pi (D_{1}^{2}-D_{2}^{2})l_{s}}{4}$$

where $V_{4}$ is the initial volume of the rubber wall of the forward artificial muscle, $D_{1}$ and $D_{2}$ are its initial outer and inner diameters, respectively, and $l_{s}$ is its effective length before deformation.

The volume of a single artificial muscle after the deformation of the forward actuator is given by

(4) $$V_{4}=\frac{\pi (D_{1}^{2}-D\prime_{2}^{2})(l_{s}+\Delta l_{4})}{4}$$

where $\Delta l_{4}$ is the axial elongation of the forward artificial muscle.

By combining Equations (3) and (4), the cross-sectional area of the artificial muscle after deformation is given by

(5) $$S\prime_{4}=\frac{\pi (D_{1}^{2}\Delta l_{4}+D_{2}^{2}l_{s})}{4(l_{s}+\Delta l_{4})}$$

When the air pressure of the forward actuator is P4, the bending angle of the VSD flexible bending joint is θ. Based on the geometric relationship of joint deformation (Figure 3c), the bending curvature radius of the elastic steel plate is given by

(6) $$\rho =\frac{l_{s}}{\theta }$$

where ρ is the radius of curvature of the neutral axis of the elastic steel plate.

The bending curvature radius of the forward actuator is given by

(7) $$\rho_{4}=\rho +L_{4}=\frac{l_{s}}{\theta }+L_{4}$$

where $\rho_{4}$ is the radius of curvature of the layer containing the forward actuator and forward VSD, and $L_{4}$ is the distance from this layer to the neutral axis of the elastic steel plate.

The bending curvature radius of the reverse actuator is given by

(8) $$\rho_{5}=\rho -L_{5}=\frac{l_{s}}{\theta }-L_{5}$$

where $\rho_{5}$ is the radius of curvature of the layer containing the reverse actuator and reverse VSD, and $L_{5}$ is the distance from this layer to the neutral axis of the elastic steel plate.

The elongation of the forward actuator is given by

(9) $$\Delta l_{4}=\rho_{4}\theta -l_{s}=L_{4}\theta$$

Assuming uniform deformation of the artificial muscles, the distances from the central axes of the forward and reverse artificial muscles to the centerline of the elastic steel plate are equal. During forward bending of the joint, the gaps between adjacent constraint rings prevent the artificial muscles from being completely constrained in the radial direction. Consequently, local radial expansion occurs at these gaps, increasing the cross-sectional area of the artificial-muscle cavity. Therefore, the driving-force correction coefficient $K_{p}$, experimentally determined by comparing the measured output force of the self-developed artificial muscle with its theoretical driving force, is introduced. According to classical elasticity theory [33], the following expression is obtained:

(10) $$M_{4}=\frac{K_{p}\pi P_{4}(D_{1}^{2}L_{x}\theta +D_{2}^{2}l_{s})L_{x}}{4(l_{s}+L_{x}\theta )}$$

According to engineering mechanics and mechanics of materials [34,35], the resisting moment generated during the bending deformation process of the elastic steel plate is given by

(11) $$M_{T}=\frac{E_{T}I_{T}}{\rho }=\frac{E_{T}bt^{3}\theta }{12l_{s}(1-\mu^{2})}$$

where $E_{T}$ and $I_{T}$ are the elastic modulus and second moment of area of the elastic steel plate, respectively; b and t are the width and thickness of the elastic steel plate, respectively; and μ is Poisson’s ratio.

The resisting moment generated by a single artificial muscle of the forward actuator is given by

(12) $$M_{r4}=F_{r4}L_{x}+M_{n}$$

According to the classical theory of elasticity, the axial deformation-resisting force of a single artificial muscle of the forward actuator is given by

(13) $$F_{r4}=\frac{E\pi ({D_{1}}^{2}-{D_{2}}^{2})l_{s}L_{x}\theta }{4{\left(l_{s}+L_{x}\theta \right)}^{2}}=\frac{E\pi ({D_{1}}^{2}-{D_{2}}^{2})l_{s}\Delta l_{4}}{4{\left(l_{s}+\Delta l_{4}\right)}^{2}}$$

where $F_{r4}$ is the axial deformation-resisting force of the forward artificial muscle, and E is the elastic modulus of the artificial-muscle material.

The bending moment generated by the forward actuator bending around its body is given by

(14) $$M_{n}=\frac{E\pi (D_{1}^{2}-D_{2}^{2})\left[(D_{1}^{2}+D_{2}^{2}){l_{s}}^{2}+2D_{1}^{2}l_{s}\Delta l_{4}\right]\theta }{64{\left(l_{s}+\Delta l_{4}\right)}^{3}}$$

where $M_{n}$ is the bending moment generated by the forward actuator bending about its own central axis.

Therefore, the deformation-resisting moment of the forward artificial muscle is given by

(15) $$M_{r4}=\frac{E\pi ({D_{1}}^{2}-{D_{2}}^{2})l_{s}L_{x}^{2}\theta }{4{\left(l_{s}+L_{x}\theta \right)}^{2}}+\frac{E\pi ({D_{1}}^{2}-{D_{2}}^{2})\left[({D_{1}}^{2}+{D_{2}}^{2})l_{s}^{2}+2{D_{1}}^{2}l_{s}L_{x}\theta \right]\theta }{64{\left(l_{s}+L_{x}\theta \right)}^{3}}$$

When the forward actuator is pressurized and the VSD flexible bending joint bends forward, the reverse actuator is subjected to compressive stress. Since the artificial muscle is a hyperelastic material, its deformation can be regarded as tension–compression bar deformation under the action of the constraint rings. Considering the large deformation of the artificial muscle, the axial deformation resisting force of the reverse artificial muscle is given by

(16) $$F_{r4}=\frac{E\pi ({D_{1}}^{2}-{D_{2}}^{2})l_{s}L_{x}\theta }{4{\left(l_{s}-L_{x}\theta \right)}^{2}}$$

Therefore, the resisting moment of the reverse artificial muscle is given by

(17) $$M_{r5}=\frac{E\pi (D_{1}^{2}-D_{2}^{2})l_{s}{L_{x}}^{2}\theta }{4{\left(l_{s}-L_{x}\theta \right)}^{2}}+\frac{E\pi (D_{1}^{2}-D_{2}^{2})\left[(D_{1}^{2}+D_{2}^{2}){l_{s}}^{2}-2D_{1}^{2}l_{s}L_{x}\theta \right]\theta }{64{\left(l_{s}-L_{x}\theta \right)}^{3}}$$

When the forward variable stiffness device is filled with pressurized gas, the driving moment generated on the upper end cap of the VSD flexible bending joint is given by

(18) $$M_{a4}={F_{P}}_{d}L_{x}=\frac{K_{A}P_{4}(\pi D_{L2}^{2}L_{x}\theta +\pi D_{c2}^{2}l_{s})L_{x}}{4(l_{s}+L_{x}\theta )}$$

where $F_{Pd}$ is the pressure-induced force acting on the upper end cap of the forward VSD.

When the VSD flexible bending joint bends forward, the resisting moment of the forward VSD includes the deformation-resistance moment $M_{x}$ generated about its own axis and the deformation-resistance moment Mat generated about the elastic steel plate.

(19) $$M_{ra}=M_{x}+M_{at}$$

where $M_{x}$ is the deformation-resistance moment of the forward VSD about its own axis, and $M_{at}$ is its deformation-resistance moment about the elastic steel plate.

When the flexible bending joint undergoes forward deformation, the forward variable stiffness device is in tension, resulting in no extrusion force. Consequently, the deformation-resistance moment $M_{x}$ generated by the forward variable stiffness device about its own axis is given by

(20) $$\begin{array}{l} M_{x}=\frac{E\pi \left(D_{c1}-D_{c2}\right)\left[\left(D_{c1}+D_{c2}\right)l_{s}^{2}+2D_{L2}l_{s}L_{x}\theta \right]\theta }{64{\left(l_{s}+L_{x}\theta \right)}^{3}}+\frac{E\pi \left(D_{y1}-D_{y2}\right)\left[\left(D_{y1}+D_{y2}\right)l_{s}^{2}+2D_{y1}l_{s}L_{x}\theta \right]\theta }{64{\left(l_{s}+L_{x}\theta \right)}^{3}}\\ \left[\begin{array}{l} fD_{L2}+nf_{2}\left(\sqrt{\frac{{D_{c1}}^{2}l_{s}+{D_{L2}}^{2}L_{x}\theta }{l_{s}+L_{x}\theta }}+\sqrt{\frac{{D_{y1}}^{2}L_{x}\theta +{D_{y2}}^{2}l_{s}}{l_{s}+L_{x}\theta }}\right)\\ +nf_{3}\sqrt{\frac{{D_{y1}}^{2}L_{x}\theta +{D_{y2}}^{2}l_{s}}{l_{s}+L_{x}\theta }}+nf_{4}\sqrt{\frac{{D_{c1}}^{2}l_{s}+{D_{L2}}^{2}L_{x}\theta }{l_{s}+L_{x}\theta }} \end{array}\right]P_{4}\pi \sqrt{\left({D_{L2}}^{2}L_{x}\theta +{D_{c2}}^{2}l_{s}\right)\left(l_{s}+L_{x}\theta \right)} \end{array}$$

The deformation-resistance moment Mat of the forward variable stiffness device about the elastic steel plate is given by

(21) $$M_{at}=\left(\sum_{i=1}^{4}F_{fi}+F_{ry}+F_{rc}\right)L_{x}$$

where $F_{fi}$ is the internal friction force of the forward VSD, and $F_{ry}$ and $F_{rc}$ are the deformation-resistance forces generated by the outer and inner air chambers during elongation, respectively.

It is known that

(22) $$\sum_{i=1}^{4}F_{fi}=(f_{1}+nf_{2}+nf_{3}+nf_{4})P_{4}\pi \sqrt{\left(D_{L2}^{2}L_{x}\theta +D_{c2}^{2}l_{s}\right)\left(l_{s}+L_{x}\theta \right)}$$

(23) $$F_{ry}=\frac{E\pi (D_{y1}^{2}-D_{y2}^{2})l_{s}L_{x}\theta }{4{\left(l_{s}+L_{x}\theta \right)}^{2}}$$

(24) $$F_{rc}=\frac{E\pi (D_{c1}^{2}-D_{c2}^{2})l_{s}L_{x}\theta }{4{\left(l_{s}+L_{x}\theta \right)}^{2}}$$

Substituting Equations (22) and (23) into Equation (21) yields

(25) $$\begin{array}{l} M_{at}=\frac{E\pi \left[\left(D_{y1}^{2}-D_{y2}^{2})+(D_{c1}^{2}-D_{c2}^{2}\right)\right]l_{s}{L^{2}}_{x}\theta }{4{\left(l_{s}+L_{x}\theta \right)}^{2}}\\ +(f_{1}+nf_{2}+nf_{3}+nf_{4})P_{4}\pi L_{x}\sqrt{\left(D_{L2}^{2}L_{x}\theta +D_{c2}^{2}l_{s}\right)\left(l_{s}+L_{x}\theta \right)} \end{array}$$

Substituting Equations (20) and (25) into Equation (19) yields

(26) $$\begin{array}{l} M_{ra}=\frac{E\pi (D_{y1}^{2}-D_{y2}^{2})\left[(D_{y1}^{2}+D_{y2}^{2}){l_{s}}^{2}+2D_{y1}^{2}l_{s}L_{x}\theta \right]\theta }{64{\left(l_{s}+L_{x}\theta \right)}^{3}}\\ +\frac{E\pi (D_{c1}^{2}-D_{c2}^{2})\left[(D_{c1}^{2}+D_{c2}^{2}){l_{s}}^{2}+2D_{L2}^{2}l_{s}L_{x}\theta \right]\theta }{64{\left(l_{s}+L_{x}\theta \right)}^{3}}+\frac{E\pi \left[\left(D_{y1}^{2}-D_{y2}^{2})+(D_{c1}^{2}-D_{c2}^{2}\right)\right]l_{s}{L^{2}}_{x}\theta }{4{\left(l_{s}+L_{x}\theta \right)}^{2}}\\ +(f_{1}+nf_{2}+nf_{3}+nf_{4})P_{4}\pi L_{x}\sqrt{\left(D_{L2}^{2}L_{x}\theta +D_{c2}^{2}l_{s}\right)\left(l_{s}+L_{x}\theta \right)}\\ +\left[f_{1}D_{L2}+\left(nf_{2}+nf_{3}\right)\sqrt{\frac{D_{y1}^{2}L_{x}\theta +D_{y2}^{2}l_{s}}{l_{s}+L_{x}\theta }}+\left(nf_{2}+nf_{4}\right)\sqrt{\frac{D_{L2}^{2}L_{x}\theta +D_{c1}^{2}l_{s}}{l_{s}+L_{x}\theta }}\right]\\ P_{4}\pi \sqrt{\left(D_{L2}^{2}L_{x}\theta +D_{c2}^{2}l_{s}\right)\left(l_{s}+L_{x}\theta \right)} \end{array}$$

Under the action of the driving moment, the unpressurized reverse variable stiffness device is passively compressed. Because the flexible inner and outer air chambers deform with the compression, the internal friction on this side is relatively small and is therefore neglected. In contrast, particle squeezing in the pressurized forward variable stiffness device generates frictional resistance, which contributes to the deformation resistance moment. In the compressed state, the available volume of the embedding grooves in the gap constraint rings is small; therefore, no appreciable extrusion force is generated between the particles and the outer air chamber. Consequently, the resisting moment of the reverse variable stiffness device consists only of the deformation resistance moments of the inner and outer air chambers.

(27) $$M_{rb}=M_{ry}+M_{rc}$$

(28) $$M_{ry}=\frac{E\pi (D_{y1}^{2}-D_{y2}^{2})l_{s}{L^{2}}_{x}\theta }{4{\left(l_{s}-L_{x}\theta \right)}^{2}}+\frac{E\pi (D_{y1}^{2}-D_{y2}^{2})\left[(D_{y1}^{2}+D_{y2}^{2}){l_{s}}^{2}-2D_{y1}^{2}l_{s}L_{x}\theta \right]}{64{\left(l_{s}-L_{x}\theta \right)}^{3}}$$

(29) $$M_{rc}=\frac{E\pi (D_{c1}^{2}-D_{c2}^{2})l_{s}{L^{2}}_{x}\theta }{4{\left(l_{s}-L_{x}\theta \right)}^{2}}+\frac{E\pi (D_{c1}^{2}-D_{c2}^{2})\left[(D_{c1}^{2}+D_{c2}^{2}){l_{s}}^{2}-2D_{L2}^{2}l_{s}L_{x}\theta \right]}{64{\left(l_{s}-L_{x}\theta \right)}^{3}}$$

Substituting Equations (28) and (29) into Equation (27) yields

(30) $$\begin{array}{l} M\prime_{rb}=\frac{E\pi \left[\left(D_{y1}^{2}-D_{y2}^{2})+(D_{c1}^{2}-D_{c2}^{2}\right)\right]l_{s}{L^{2}}_{x}\theta }{4{\left(l_{s}-L_{x}\theta \right)}^{2}}\\ +\frac{E\pi (D_{c1}^{2}-D_{c2}^{2})\left[(D_{c1}^{2}+D_{c2}^{2}){l_{s}}^{2}-2D_{L2}^{2}l_{s}L_{x}\theta \right]}{64{\left(l_{s}-L_{x}\theta \right)}^{3}}\\ +\frac{E\pi (D_{y1}^{2}-D_{y2}^{2})\left[(D_{y1}^{2}+D_{y2}^{2}){l_{s}}^{2}-2D_{y1}^{2}l_{s}L_{x}\theta \right]}{64{\left(l_{s}-L_{x}\theta \right)}^{3}} \end{array}$$

The outer air chamber of the variable stiffness device and the elastic airbags of the forward (reverse) artificial muscles adopt rubber hoses with the same specifications. Therefore, substituting Equations (10), (12), (15), (17), (26) and (30) into Equation (1), the relationship between the bending angle and air pressure of the flexible bending joint can be obtained.

(31) $$\begin{array}{l} f(P_{4},\theta )=\frac{K_{P}\pi P_{4}(D_{1}^{2}L_{x}\theta +D_{2}^{2}l_{s})L_{x}}{2(l_{s}+L_{x}\theta )}+\frac{K_{A}\pi P_{4}(D_{L1}^{2}L_{x}\theta +D_{c2}^{2}l_{s})L_{x}}{4(l_{s}+L_{x}\theta )}-\frac{E_{b}bt^{3}\theta }{12l_{s}(1-\mu^{2})}\\ -\frac{E\pi \left[3(D_{1}^{2}-D_{2}^{2})+(D_{c1}^{2}-D_{c2}^{2})\right]l_{s}{L^{2}}_{x}\theta }{4{\left(l_{s}+L_{x}\theta \right)}^{2}}-\frac{3E\pi (D_{1}^{2}-D_{2}^{2})\left[(D_{1}^{2}+D_{2}^{2})l_{s}+2D_{1}^{2}l_{s}L_{x}\theta \right]\theta }{64{\left(l_{s}+L_{x}\theta \right)}^{3}}\\ -\frac{E\pi \left[3(D_{1}^{2}-D_{2}^{2})+(D_{c1}^{2}-D_{c2}^{2})\right]l_{s}{L^{2}}_{x}\theta }{4{\left(l_{s}-L_{x}\theta \right)}^{2}}-\frac{3E\pi (D_{1}^{2}-D_{2}^{2})\left[(D_{1}^{2}+D_{2}^{2})l_{s}-2D_{1}^{2}l_{s}L_{x}\theta \right]\theta }{64{\left(l_{s}-L_{x}\theta \right)}^{3}}\\ -\frac{E\pi (D_{c1}^{2}-D_{c2}^{2})\left[(D_{c1}^{2}+D_{c2}^{2}){l_{s}}^{2}+2D_{L2}^{2}l_{s}L_{x}\theta \right]\theta }{64{\left(l_{s}+L_{x}\theta \right)}^{3}}-\frac{E\pi (D_{c1}^{2}-D_{c2}^{2})\left[(D_{c1}^{2}+D_{c2}^{2}){l_{s}}^{2}-2D_{L2}^{2}l_{s}L_{x}\theta \right]\theta }{64{\left(l_{s}-L_{x}\theta \right)}^{3}}\\ -(f_{1}+nf_{2}+nf_{3}+nf_{4})P_{4}\pi L_{x}\sqrt{\left(D_{L2}^{2}L_{x}\theta +D_{c2}^{2}l_{s}\right)\left(l_{s}+L_{x}\theta \right)}\\ -\left[\begin{array}{l} f_{1}D_{L2}+\left(nf_{2}+nf_{3}\right)\sqrt{\frac{D_{y1}^{2}L_{x}\theta +D_{y2}^{2}l_{s}}{l_{s}+L_{x}\theta }}\\ +\left(nf_{2}+nf_{4}\right)\sqrt{\frac{D_{L2}^{2}L_{x}\theta +D_{c1}^{2}l_{s}}{l_{s}+L_{x}\theta }} \end{array}\right]P_{4}\pi \sqrt{\left(D_{L2}^{2}L_{x}\theta +D_{c2}^{2}l_{s}\right)\left(l_{s}+L_{x}\theta \right)} \end{array}$$

### 3.3. Tangential Stiffness Along the Bending Direction

**Figure 4:**
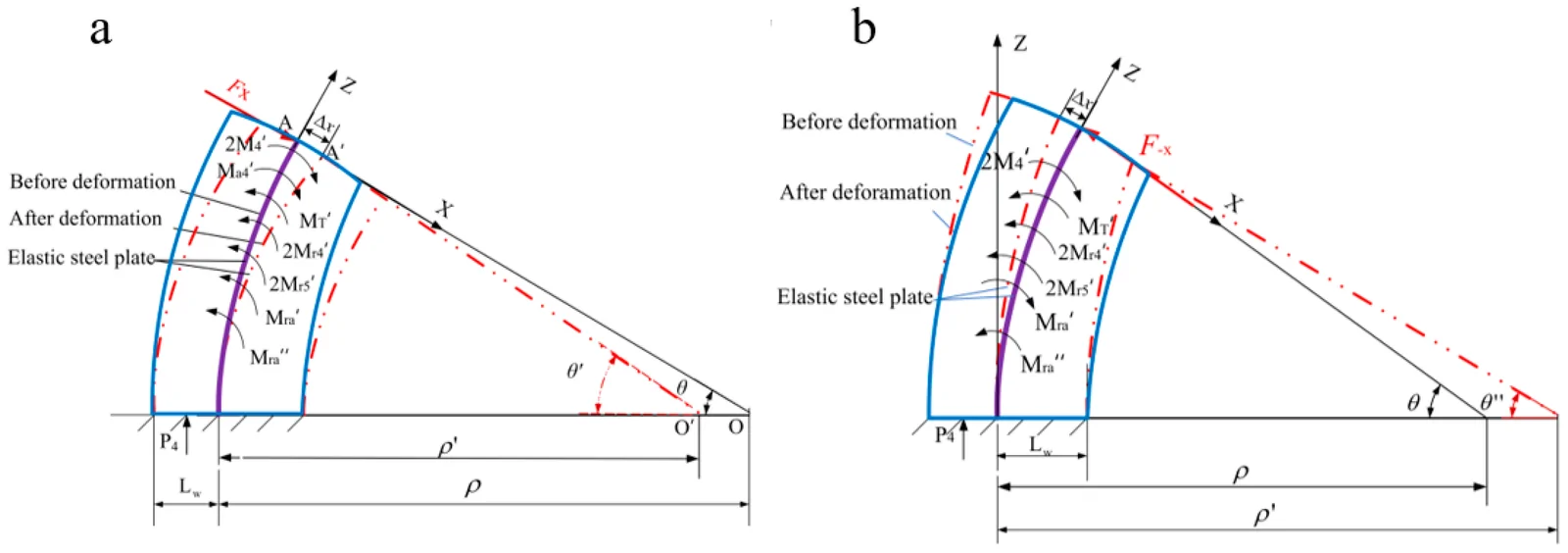
Mechanical models of the flexible bending joint under tangential loading: (**a**) forward loading under $F_{x}$; (**b**) reverse loading under $F_{-x}$.

Taking forward deformation as an example, the force-bending model of the joint is established (Figure 4a). The pressures applied to the forward actuator and forward VSD are both P4, and the initial bending angle of the joint is θ. When an external force $F_{x}$ along the positive *x*-axis is applied to the upper end cap, the bending angle of the flexible bending joint changes to θ’.

After the joint undergoes bending deformation, the air pressure of the artificial muscles and variable stiffness devices remains constant. A slight deformation occurs under external force disturbance, and the cross-sectional area of the inner air chamber cavity is regarded as unchanged. Therefore

(32) $$F_{x}l_{x}+2M\prime_{4}+M\prime_{a4}=M\prime_{T}+2M\prime_{r4}+2M\prime_{r5}+M\prime_{ra}+M\prime_{rb}$$

where $M\prime_{4}$, $M\prime_{T}$, and $M\prime_{r4}$ are the driving moment of the forward actuator, the resisting moment of the elastic steel plate, and the resisting moment of the forward actuator after the joint angle changes from θ to θ′, respectively; ρ′ is the corresponding radius of curvature of the neutral axis of the elastic steel plate.

The external force $F_{x}$ acts on the geometric center of the upper end cap of the flexible bending joint, so its moment arm is the length of the elastic steel plate. The elastic steel plate is inextensible; hence, the moment arm of $F_{x}$ is

(33) $$l_{x}=l_{s}$$

Based on the aforementioned constant pressure boundary assumption, it can be obtained that

(34) $$M\prime_{4}=\frac{K_{P}\pi P_{4}(D_{1}^{2}L_{x}\theta +D_{2}^{2}l_{s})L_{x}}{4(l_{s}+L_{x}\theta )}$$

(35) $$M\prime_{T}=\frac{E_{b}bt^{3}\theta^{\prime }}{12l_{s}(1-\mu^{2})}$$

(36) $$M\prime_{r4}=\frac{E\pi ({D_{1}}^{2}-{D_{2}}^{2})l_{s}L_{x}^{2}\theta^{\prime }}{4{\left(l_{s}+L_{x}\theta^{\prime }\right)}^{2}}+\frac{E\pi ({D_{1}}^{2}-{D_{2}}^{2})\left[({D_{1}}^{2}+{D_{2}}^{2})l_{s}^{2}+2{D_{1}}^{2}l_{s}L_{x}\theta^{\prime }\right]\theta^{\prime }}{64{\left(l_{s}+L_{x}\theta^{\prime }\right)}^{3}}$$

(37) $$M\prime_{r5}=\frac{E\pi ({D_{1}}^{2}-{D_{2}}^{2})l_{s}L_{x}^{2}\theta^{\prime }}{4{\left(l_{s}-L_{x}\theta^{\prime }\right)}^{2}}+\frac{E\pi ({D_{1}}^{2}-{D_{2}}^{2})\left[({D_{1}}^{2}+{D_{2}}^{2})l_{s}^{2}-2{D_{1}}^{2}l_{s}L_{x}\theta^{\prime }\right]\theta^{\prime }}{64{\left(l_{s}-L_{x}\theta^{\prime }\right)}^{3}}$$

(38) $$M\prime_{a4}=\frac{K_{A}P_{4}(\pi D_{L2}^{2}L_{x}\theta +\pi D_{c2}^{2}l_{s})L_{x}}{4(l_{s}+L_{x}\theta )}$$

(39) $$\begin{array}{l} M\prime_{ra}=\frac{E\pi (D_{y1}^{2}-D_{y2}^{2})\left[(D_{y1}^{2}+D_{y2}^{2}){l_{s}}^{2}+2D_{y1}^{2}l_{s}L_{x}\theta^{\prime }\right]\theta^{\prime }}{64{\left(l_{s}+L_{x}\theta^{\prime }\right)}^{3}}\\ +\frac{E\pi (D_{c1}^{2}-D_{c2}^{2})\left[(D_{c1}^{2}+D_{c2}^{2}){l_{s}}^{2}+2D_{L2}^{2}l_{s}L_{x}\theta^{\prime }\right]\theta^{\prime }}{64{\left(l_{s}+L_{x}\theta^{\prime }\right)}^{3}}+\frac{E\pi \left[\left(D_{y1}^{2}-D_{y2}^{2})+(D_{c1}^{2}-D_{c2}^{2}\right)\right]l_{s}{L^{2}}_{x}\theta^{\prime }}{4{\left(l_{s}+L_{x}\theta^{\prime }\right)}^{2}}\\ +(f_{1}+nf_{2}+nf_{3}+nf_{4})P_{4}\pi L_{x}\sqrt{\left(D_{L2}^{2}L_{x}\theta +D_{c2}^{2}l_{s}\right)\left(l_{s}+L_{x}\theta \right)}\\ +\left[f_{1}D_{L2}+\left(nf_{2}+nf_{3}\right)\sqrt{\frac{D_{y1}^{2}L_{x}\theta +D_{y2}^{2}l_{s}}{l_{s}+L_{x}\theta }}+\left(nf_{2}+nf_{4}\right)\sqrt{\frac{D_{L2}^{2}L_{x}\theta +D_{c1}^{2}l_{s}}{l_{s}+L_{x}\theta }}\right]\\ P_{4}\pi \sqrt{\left(D_{L2}^{2}L_{x}\theta +D_{c2}^{2}l_{s}\right)\left(l_{s}+L_{x}\theta \right)} \end{array}$$

(40) $$\begin{array}{l} M{\prime \prime }_{ra}=\frac{E\pi \left[\left(D_{y1}^{2}-D_{y2}^{2})+(D_{c1}^{2}-D_{c2}^{2}\right)\right]l_{s}{L^{2}}_{x}\theta \prime }{4{\left(l_{s}-L_{x}\theta \prime \right)}^{2}}+\frac{E\pi (D_{c1}^{2}-D_{c2}^{2})\left[(D_{c1}^{2}+D_{c2}^{2}){l_{s}}^{2}-2D_{L2}^{2}l_{s}L_{x}\theta \prime \right]\theta \prime }{64{\left(l_{s}-L_{x}\theta \prime \right)}^{3}}\\ +\frac{E\pi (D_{y1}^{2}-D_{y2}^{2})\left[(D_{y1}^{2}+D_{y2}^{2}){l_{s}}^{2}+2D_{y1}^{2}l_{s}L_{x}\theta \prime \right]\theta \prime }{64{\left(l_{s}-L_{x}\theta \prime \right)}^{3}} \end{array}$$

The relationship between the bending angle θ’ and the external load $F_{x}$ is

(41) $$\begin{array}{l} f(F_{x},\theta \prime )=F_{x}l_{s}+\frac{K_{P}\pi P_{4}(D_{1}^{2}L_{x}\theta +D_{2}^{2}l_{s})L_{x}}{2(l_{s}+L_{x}\theta )}+\frac{K_{A}\pi P_{4}(D_{L1}^{2}L_{x}\theta +D_{c2}^{2}l_{s})L_{x}}{4(l_{s}+L_{x}\theta )}\\ -\frac{E_{b}bt^{3}\theta^{\prime }}{12l_{s}(1-\mu^{2})}-\frac{E\pi \left[3(D_{1}^{2}-D_{2}^{2})+(D_{c1}^{2}-D_{c2}^{2})\right]l_{s}{L^{2}}_{x}\theta^{\prime }}{4{\left(l_{s}+L_{x}\theta^{\prime }\right)}^{2}}\\ -\frac{3E\pi (D_{1}^{2}-D_{2}^{2})\left[(D_{1}^{2}+D_{2}^{2})l_{s}+2D_{1}^{2}l_{s}L_{x}\theta^{\prime }\right]\theta^{\prime }}{64{\left(l_{s}+L_{x}\theta^{\prime }\right)}^{3}}-\frac{E\pi \left[3(D_{1}^{2}-D_{2}^{2})+(D_{c1}^{2}-D_{c2}^{2})\right]l_{s}{L^{2}}_{x}\theta^{\prime }}{4{\left(l_{s}-L_{x}\theta^{\prime }\right)}^{2}}\\ -\frac{3E\pi (D_{1}^{2}-D_{2}^{2})\left[(D_{1}^{2}+D_{2}^{2})l_{s}-2D_{1}^{2}l_{s}L_{x}\theta^{\prime }\right]\theta^{\prime }}{64{\left(l_{s}-L_{x}\theta^{\prime }\right)}^{3}}-\frac{E\pi (D_{c1}^{2}-D_{c2}^{2})\left[(D_{c1}^{2}+D_{c2}^{2}){l_{s}}^{2}+2D_{L2}^{2}l_{s}L_{x}\theta^{\prime }\right]\theta^{\prime }}{64{\left(l_{s}+L_{x}\theta^{\prime }\right)}^{3}}\\ -\frac{E\pi (D_{c1}^{2}-D_{c2}^{2})\left[(D_{c1}^{2}+D_{c2}^{2}){l_{s}}^{2}-2D_{L2}^{2}l_{s}L_{x}\theta^{\prime }\right]\theta^{\prime }}{64{\left(l_{s}-L_{x}\theta^{\prime }\right)}^{3}}\\ -(f_{1}+nf_{2}+nf_{3}+nf_{4})P_{4}\pi L_{x}\sqrt{\left(D_{L2}^{2}L_{x}\theta +D_{c2}^{2}l_{s}\right)\left(l_{s}+L_{x}\theta \right)}\\ -\left[\begin{array}{l} f_{1}D_{L2}+\left(nf_{2}+nf_{3}\right)\sqrt{\frac{D_{y1}^{2}L_{x}\theta +D_{y2}^{2}l_{s}}{l_{s}+L_{x}\theta }}\\ +\left(nf_{2}+nf_{4}\right)\sqrt{\frac{D_{L2}^{2}L_{x}\theta +D_{c1}^{2}l_{s}}{l_{s}+L_{x}\theta }} \end{array}\right]P_{4}\pi \sqrt{\left(D_{L2}^{2}L_{x}\theta +D_{c2}^{2}l_{s}\right)\left(l_{s}+L_{x}\theta \right)} \end{array}$$

Therefore, under the above deformation conditions, the stiffness of the joint along the bending direction is

(42) $$K_{wx}=\frac{F_{x}}{\Delta x}=\frac{F_{x}\theta \theta^{\prime }\sin\theta }{l_{s}(\theta^{\prime }\sin\theta -\theta \sin\theta^{\prime })}$$

where $k_{t}$ is the tangential stiffness along the bending direction, and $F_{x}$ is the external force applied along the positive x-axis.

### 3.4. Tangential Stiffness Along the Reverse Bending Direction

Still taking forward deformation as an example, the force–bending model of the joint is established (Figure 4b). The pressures applied to the forward actuator and forward VSD are both P4, and the initial bending angle of the joint is θ. An external force $F_{-x}$ is then applied to the upper end cap along the negative x−axis, causing the bending angle of the joint to change to θ″.

According to the static equilibrium equation, it is obtained that

(43) $$2M\prime_{4}+M\prime_{ra}=F_{-x}l_{s}+M\prime_{T}+2M\prime_{r4}+2M\prime_{r5}+M{\prime \prime }_{ra}$$

The air pressure inside the joint remains constant throughout. Accordingly, when the elongation decreases under external force, the artificial muscle is compressed and the cross-sectional area of the inner cavity changes. Therefore

(44) $$M\prime_{4}=\frac{K_{P}\pi P_{4}(D_{1}^{2}L_{x}\theta^{\prime \prime }+D_{2}^{2}l_{s})L_{x}}{4(l_{s}+L_{x}\theta^{\prime \prime })}$$

(45) $$M\prime_{T}=\frac{E_{b}bt^{3}\theta^{\prime \prime }}{12l_{s}(1-\mu^{2})}$$

(46) $$M\prime_{r4}=\frac{E\pi ({D_{1}}^{2}-{D_{2}}^{2})l_{s}L_{x}^{2}\theta^{\prime }}{4{\left(l_{s}+L_{x}\theta^{\prime }\right)}^{2}}+\frac{E\pi ({D_{1}}^{2}-{D_{2}}^{2})\left[({D_{1}}^{2}+{D_{2}}^{2})l_{s}^{2}+2{D_{1}}^{2}l_{s}L_{x}\theta^{\prime }\right]\theta^{\prime }}{64{\left(l_{s}+L_{x}\theta^{\prime }\right)}^{3}}$$

(47) $$M\prime_{r5}=\frac{E\pi ({D_{1}}^{2}-{D_{2}}^{2})l_{s}L_{x}^{2}\theta^{\prime }}{4{\left(l_{s}-L_{x}\theta^{\prime }\right)}^{2}}+\frac{E\pi ({D_{1}}^{2}-{D_{2}}^{2})\left[({D_{1}}^{2}+{D_{2}}^{2})l_{s}^{2}-2{D_{1}}^{2}l_{s}L_{x}\theta^{\prime }\right]\theta^{\prime }}{64{\left(l_{s}-L_{x}\theta^{\prime }\right)}^{3}}$$

(48) $$M\prime_{a4}=\frac{K_{A}P_{4}(\pi D_{L2}^{2}L_{x}\theta^{\prime \prime }+\pi D_{c2}^{2}l_{s})L_{x}}{4(l_{s}+L_{x}\theta^{\prime \prime })}$$

When subjected to the external force $F_{-x}$, the moment generated by the airbag in the forward variable stiffness device, the friction moment, as well as the extrusion moments on the tension and compression sides of the gap constraint rings, are all opposite to the moment of $F_{-x}$. Meanwhile, the resistance moments of the inner air chamber and outer air chamber are in the same direction as the moment of $F_{-x}$. Therefore, when the joint rotation angle is θ″, the resistance moments of the forward and reverse variable stiffness devices are expressed as follows, respectively:

(49) $$\begin{array}{l} M\prime_{ra}=\frac{K_{A}P_{4}\pi \left(D_{L2}^{2}L_{x}\theta^{\prime \prime }+D_{c2}^{2}l_{s}\right)L_{x}}{4\left(l_{s}+L_{x}\theta^{\prime \prime }\right)}+\frac{P_{4}\pi r^{2}\left(D_{L1}+D_{L2}\right)}{\tan(\alpha +\phi )}\\ +(f_{1}+nf_{2}+nf_{3}+nf_{4})P_{4}\pi L_{x}\sqrt{\left(D_{L2}^{2}L_{x}\theta +D_{c2}^{2}l_{s}\right)\left(l_{s}+L_{x}\theta^{\prime \prime }\right)}\\ +\left[f_{1}D_{L2}+\left(nf_{2}+nf_{3}\right)\sqrt{\frac{D_{y1}^{2}L_{x}\theta +D_{y2}^{2}l_{s}}{l_{s}+L_{x}\theta^{\prime \prime }}}+\left(nf_{2}+nf_{4}\right)\sqrt{\frac{D_{L2}^{2}L_{x}\theta^{\prime \prime }+D_{c1}^{2}l_{s}}{l_{s}+L_{x}\theta^{\prime \prime }}}\right]\\ P_{4}\pi \sqrt{\left(D_{L2}^{2}L_{x}\theta^{\prime \prime }+D_{c2}^{2}l_{s}\right)\left(l_{s}+L_{x}\theta^{\prime \prime }\right)}-\frac{E\pi \left[\left(D_{y1}^{2}-D_{y2}^{2}\right)+\left(D_{c1}^{2}-D_{c2}^{2}\right)\right]l_{s}{L_{x}}^{2}\theta^{\prime \prime }}{4{\left(l_{s}+L_{x}\theta^{\prime \prime }\right)}^{2}}\\ -\frac{E\pi \left(D_{y1}^{2}-D_{y2}^{2}\right)\left[\left(D_{y1}^{2}+D_{y2}^{2}\right){l_{s}}^{2}+2D_{y1}^{2}l_{s}L_{x}\theta^{\prime \prime }\right]\theta^{\prime \prime }}{64{\left(l_{s}+L_{x}\theta^{\prime \prime }\right)}^{3}}\\ -\frac{E\pi \left(D_{c1}^{2}-D_{c2}^{2}\right)\left[\left(D_{c1}^{2}+D_{c2}^{2}\right){l_{s}}^{2}+2D_{L2}^{2}l_{s}L_{x}\theta^{\prime \prime }\right]\theta^{\prime \prime }}{64{\left(l_{s}+L_{x}\theta^{\prime \prime }\right)}^{3}} \end{array}$$

(50) $$\begin{array}{l} M\prime_{rb}=\frac{E\pi \left[\left(D_{y1}^{2}-D_{y2}^{2})+(D_{c1}^{2}-D_{c2}^{2}\right)\right]l_{s}{L^{2}}_{x}\theta^{\prime \prime }}{4{\left(l_{s}-L_{x}\theta^{\prime \prime }\right)}^{2}}+\frac{E\pi (D_{c1}^{2}-D_{c2}^{2})\left[(D_{c1}^{2}+D_{c2}^{2}){l_{s}}^{2}-2D_{L2}^{2}l_{s}L_{x}\theta^{\prime \prime }\right]\theta^{\prime \prime }}{64{\left(l_{s}-L_{x}\theta^{\prime \prime }\right)}^{3}}\\ +\frac{E\pi (D_{y1}^{2}-D_{y2}^{2})\left[(D_{y1}^{2}+D_{y2}^{2}){l_{s}}^{2}+2D_{y1}^{2}l_{s}L_{x}\theta^{\prime \prime }\right]\theta^{\prime \prime }}{64{\left(l_{s}-L_{x}\theta^{\prime \prime }\right)}^{3}} \end{array}$$

Substituting Equations (44)–(50) into Equation (43) gives the following relationship between the bending angle θ″ and the external load $F_{-x}$:

(51) $$\begin{array}{l} f(F_{-x},\theta \prime \prime )=\frac{K_{P}\pi P_{4}(D_{1}^{2}L_{x}\theta^{\prime \prime }+D_{2}^{2}l_{s})L_{x}}{2(l_{s}+L_{x}\theta^{\prime \prime })}+\frac{K_{A}\pi P_{4}(D_{L1}^{2}L_{x}\theta^{\prime \prime }+D_{c2}^{2}l_{s})L_{x}}{4(l_{s}+L_{x}\theta^{\prime \prime })}+\frac{\pi P_{4}r^{2}\left(D_{L1}+D_{L2}\right)}{\tan(\alpha +\phi )}\\ +(f_{1}+nf_{2}+nf_{3}+nf_{4})P_{4}\pi L_{x}\sqrt{\left(D_{L2}^{2}L_{x}\theta^{\prime \prime }+D_{c2}^{2}l_{s}\right)\left(l_{s}+L_{x}\theta^{\prime \prime }\right)}\\ +\left[\begin{array}{l} f_{1}D_{L2}+\left(nf_{2}+nf_{3}\right)\sqrt{\frac{D_{y1}^{2}L_{x}\theta^{\prime \prime }+D_{y2}^{2}l_{s}}{l_{s}+L_{x}\theta^{\prime \prime }}}\\ +\left(nf_{2}+nf_{4}\right)\sqrt{\frac{D_{L2}^{2}L_{x}\theta \prime \prime +D_{c1}^{2}l_{s}}{l_{s}+L_{x}\theta^{\prime \prime }}} \end{array}\right]P_{4}\pi \sqrt{\left(D_{L2}^{2}L_{x}\theta \prime \prime +D_{c2}^{2}l_{s}\right)\left(l_{s}+L_{x}\theta^{\prime \prime }\right)}-F_{-x}l_{x}\\ -\frac{E_{b}bt^{3}\theta^{\prime \prime }}{12l_{s}(1-\mu^{2})}-\frac{E\pi \left[3(D_{1}^{2}-D_{2}^{2})+(D_{c1}^{2}-D_{c2}^{2})\right]l_{s}{L^{2}}_{x}\theta^{\prime \prime }}{4{\left(l_{s}+L_{x}\theta^{\prime \prime }\right)}^{2}}\\ -\frac{3E\pi (D_{1}^{2}-D_{2}^{2})\left[(D_{1}^{2}+D_{2}^{2})l_{s}+2D_{1}^{2}l_{s}L_{x}\theta^{\prime \prime }\right]\theta^{\prime \prime }}{64{\left(l_{s}+L_{x}\theta^{\prime \prime }\right)}^{3}}-\frac{E\pi \left[3(D_{1}^{2}-D_{2}^{2})+(D_{c1}^{2}-D_{c2}^{2})\right]l_{s}{L^{2}}_{x}\theta^{\prime \prime }}{4{\left(l_{s}-L_{x}\theta^{\prime \prime }\right)}^{2}}\\ -\frac{3E\pi (D_{1}^{2}-D_{2}^{2})\left[(D_{1}^{2}+D_{2}^{2})l_{s}-2D_{1}^{2}l_{s}L_{x}\theta^{\prime \prime }\right]\theta^{\prime \prime }}{64{\left(l_{s}-L_{x}\theta^{\prime \prime }\right)}^{3}}-\frac{E\pi (D_{c1}^{2}-D_{c2}^{2})\left[(D_{c1}^{2}+D_{c2}^{2}){l_{s}}^{2}+2D_{L2}^{2}l_{s}L_{x}\theta^{\prime \prime }\right]\theta^{\prime \prime }}{64{\left(l_{s}+L_{x}\theta^{\prime \prime }\right)}^{3}}\\ -\frac{E\pi (D_{c1}^{2}-D_{c2}^{2})\left[(D_{c1}^{2}+D_{c2}^{2}){l_{s}}^{2}-2D_{L2}^{2}l_{s}L_{x}\theta^{\prime \prime }\right]\theta^{\prime \prime }}{64{\left(l_{s}-L_{x}\theta^{\prime \prime }\right)}^{3}} \end{array}$$

Therefore, under the above deformation conditions, the stiffness of the joint in the reverse bending direction is

(52) $$K_{-wx}=\frac{F_{-x}}{\Delta x}=\frac{F_{-x}\theta \theta^{\prime \prime }\sin\theta^{\prime \prime }}{l_{s}(\theta \sin\theta^{\prime \prime }-\theta^{\prime \prime }\sin\theta )}$$

The structural and material parameters used in the theoretical model, together with their values, units, and determination methods, are summarized in Table 2.

## 4. Experiments of Flexible Bending Joints

### 4.1. Bending Angle Experiment of Flexible Bending Joint

**Figure 5:**
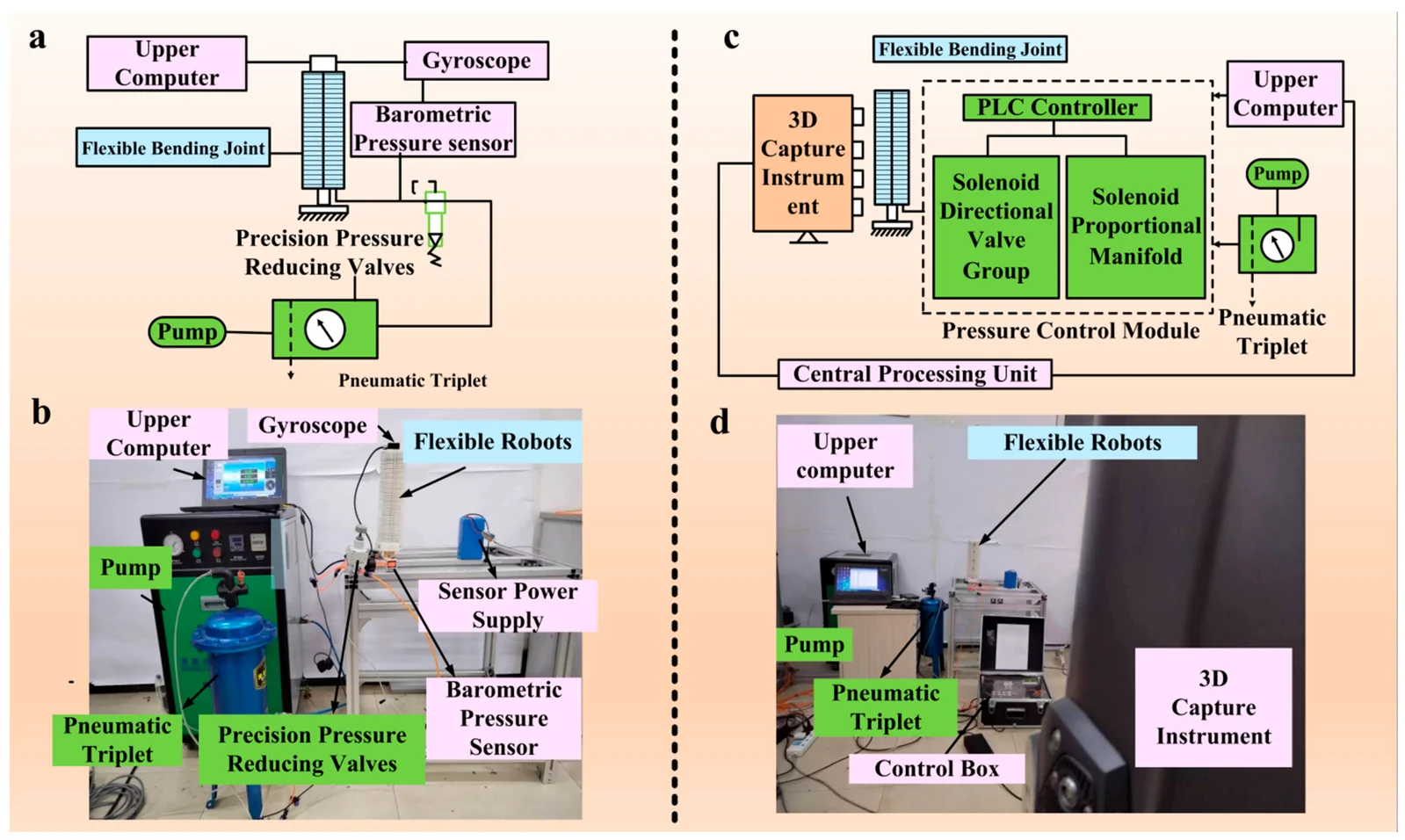
Bending experiment principle and experimental platform of the actively variable stiffness pneumatic flexible bending joint: (**a**) schematic diagram of the bending angle experiment principle for the actively variable stiffness pneumatic flexible bending joint; (**b**) bending angle experimental platform of the actively variable stiffness pneumatic flexible bending joint; (**c**) schematic diagram of the deformation principle for the actively variable stiffness pneumatic flexible bending joint; (**d**) deformation experimental platform of the actively variable stiffness pneumatic flexible bending joint.

To evaluate the motion performance of the actively variable stiffness pneumatic flexible bending joint, comparative experiments were carried out under two working conditions: without VSD and with VSD. The joint was vertically fixed on the experimental platform, and the air pressure was regulated by a pressure-reducing valve. The air pressure was increased stepwise to 0.4 MPa at an increment of 0.05 MPa to drive the joint to perform forward and reverse bending. The pressure sensor monitored the air pressure in real time, while the gyroscope synchronously collected bending-angle data, which were transmitted to the upper computer for subsequent analysis (Figure 5a). The experimental setup for the bending-performance test mainly consists of an air pump, an upper computer, a gyroscope, a flexible bending joint, a sensor power module, and an air pressure sensor (Figure 5b).

Five 3D motion capture marking points were arranged at equal intervals along the axial direction of the joint. The air pressure was increased stepwise to 0.35 MPa at an increment of 0.09 MPa to drive the joint to realize forward and reverse bending. Comparative tests were implemented under the above two working conditions. The 3D motion-capture system collected experimental data in real time and uploaded the data to the upper computer for processing (Figure 5c). The deformation-test apparatus mainly consists of a control box, an upper computer, a central processing unit, and a 3D motion-capture system (Figure 5d).

Each pressurization test was repeated five times under identical conditions over the pressure range of 0–0.4 MPa. The arithmetic mean of the five measurements at each pressure level was used as the experimental value for comparison with the theoretical prediction.

**Figure 6:**
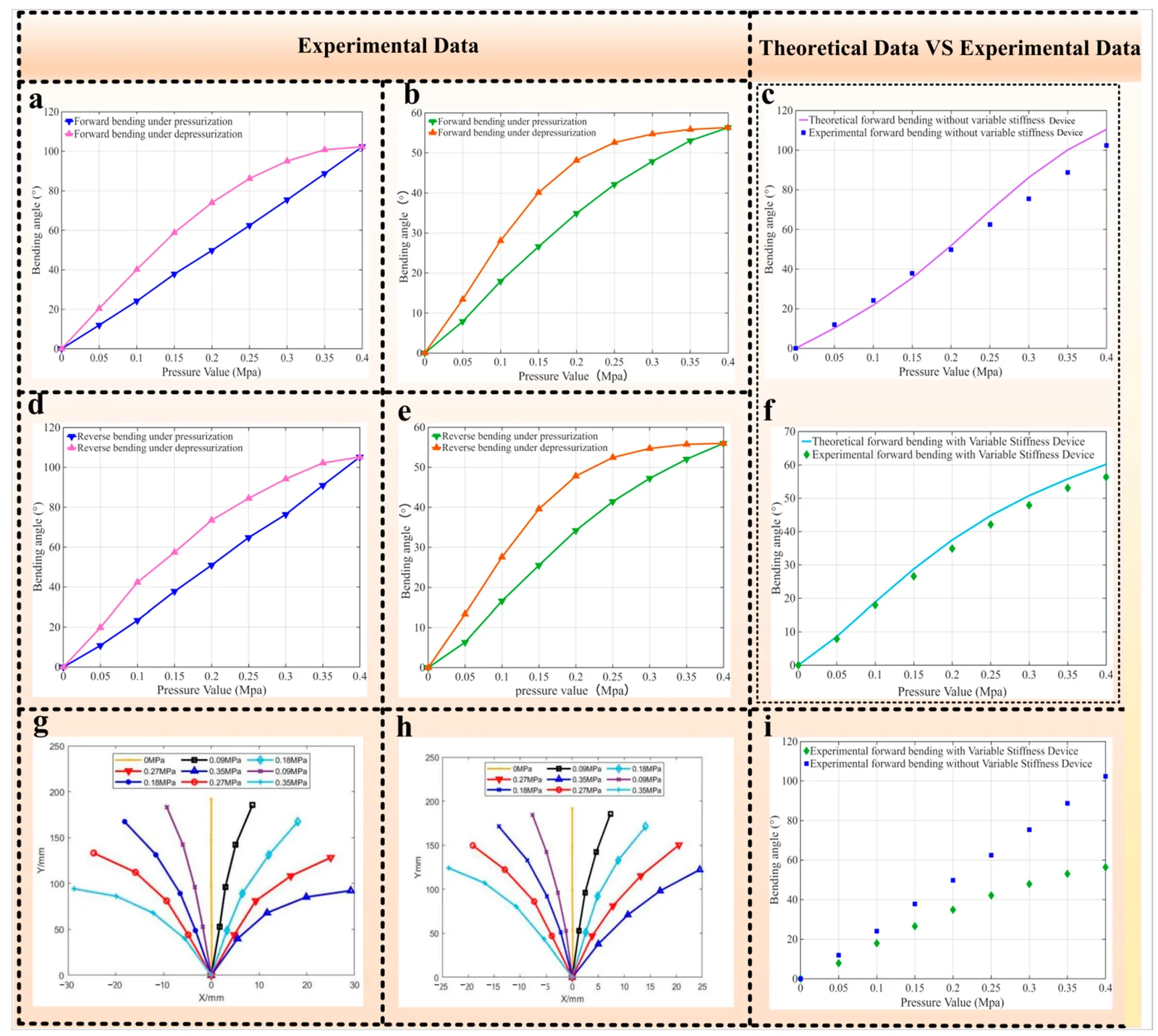
Bending experimental results of the actively variable stiffness pneumatic flexible bending joint: (**a**) forward bending experimental data of the flexible bending joint without VSD; (**b**) forward bending experimental data of the flexible bending joint with VSD; (**c**) theoretical and experimental comparison of forward bending for the flexible bending joint without VSD (the theoretical model takes forward pressurization as an example); (**d**) reverse bending experimental data of the flexible bending joint without VSD; (**e**) reverse bending experimental data of the flexible bending joint with VSD; (**f**) theoretical and experimental comparison of forward bending for the flexible bending joint with VSD; (**g**) deformation experimental data of the flexible bending joint without VSD; (**h**) deformation experimental data of the flexible bending joint with VSD; (**i**) comparative diagram of forward bending experiments for flexible bending joints with and without VSD.

The bending experimental data of the pneumatic flexible bending joints without and with VSD are shown in Figure 6a,b,d,e. Hysteresis is observed during depressurization for both joints, which is mainly attributed to the characteristics of the elastic materials. Owing to the symmetric structural design of the joint, the forward and reverse bending responses exhibit high consistency. Therefore, the forward bending results are taken as representative examples in the subsequent model validation. Without VSD, the bending angle increases nonlinearly with air pressure. The theoretical predictions are consistent with the averaged experimental results, with a mean relative error (MRE) of 10.12% (Figure 6c). The experimental bending angle reaches 102.32° at 0.4 MPa under forward bending (Figure 6a). Similarly, the bending angle of the joint with VSD increases nonlinearly with air pressure. The theoretical predictions agree with the averaged experimental results, with an MRE of 6.77% (Figure 6f), and the experimental bending angle reaches 56.35° at 0.4 MPa (Figure 6b).

Compared with the joint without VSD, the joint equipped with VSD exhibits a smaller bending angle under the same air pressure. This is mainly attributed to the bilateral arrangement of the VSDs. During bending, the inactive VSD possesses inherent structural stiffness and produces an antagonistic effect on joint deformation. The joint equipped with VSD exhibits stable overall deformation and a regular motion trajectory, indicating that the VSD configuration does not adversely affect the stability and controllability of the flexible bending joint (Figure 6g,h).

### 4.2. Tangential Stiffness Experiment of Flexible Bending Joints

**Figure 7:**
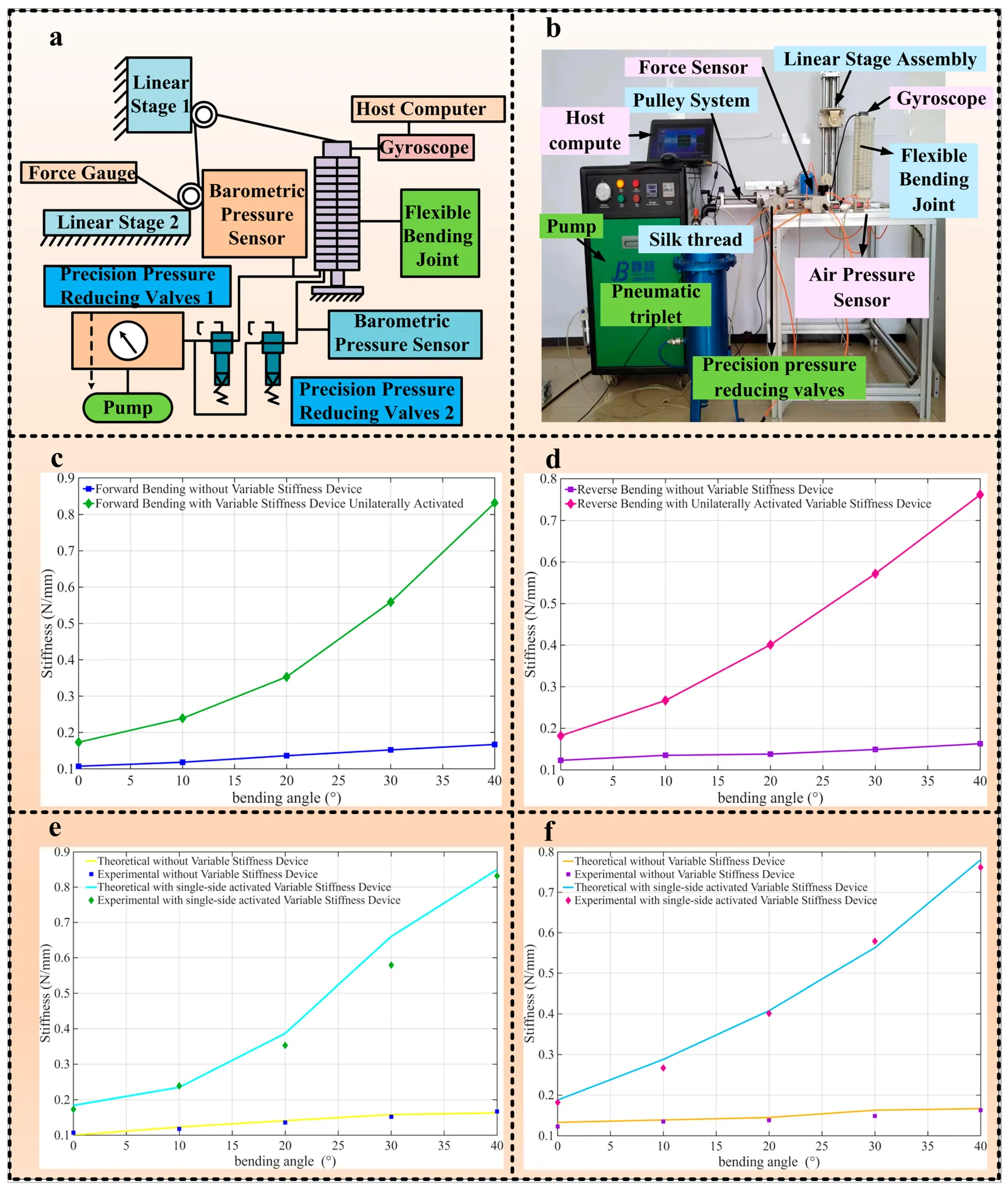
Tangential stiffness experiment of the flexible bending joint: (**a**) schematic diagram of the tangential stiffness experiment principle for the flexible bending joint; (**b**) experimental platform for tangential stiffness test of the flexible bending joint; (**c**) experimental data of tangential stiffness under forward bending of the flexible bending joint; (**d**) experimental data of tangential stiffness under reverse bending of the flexible bending joint; (**e**) comparison between theoretical predictions and experimental results of tangential stiffness under forward bending for flexible bending joints with and without VSD; (**f**) comparison between theoretical predictions and experimental results of tangential stiffness under reverse bending for flexible bending joints with and without VSD.

Two working conditions were set for comparative analysis: without VSD and with unilateral VSD activation. The test apparatus mainly consisted of a pneumatic control unit, a loading unit, a force measurement unit, a displacement measurement unit, an angle monitoring unit, and a data acquisition system (Figure 7a). In the tangential stiffness experiment, a tangential force was applied to the joint through a pulley mechanism. The corresponding tangential force was measured at bending angles ranging from 0° to 40° to evaluate the variation in tangential stiffness with bending angle (Figure 7b). The flexible bending joint was fixed on the test platform, and its distal end was connected to a double-pulley mechanism through a steel wire rope to provide a stable and direction-controllable tangential force. This experiment ensured that the distal end of the joint underwent the same displacement under all test conditions. The end displacement was monitored using a laser displacement sensor, while the corresponding tangential force was measured and recorded in real time using a digital force sensor. The tangential stiffness was subsequently determined from the measured tangential force and end displacement. The actuation pressure of the joint and the pressure of the VSD were regulated by independent pressure-reducing valves to ensure consistent loading conditions. The bending angle was measured in real time using a gyroscope. During the experiment, the host computer controlled the sliding table motion and pneumatic pressure input while synchronously recording the force, displacement, angle, and pressure data.

Each tangential stiffness experiment was repeated five times under identical experimental conditions, and the arithmetic mean of the five measurements was used as the experimental value for comparison with the theoretical prediction.

Without VSD, the forward and reverse stiffness ratios at a bending angle of 40°, relative to their corresponding values at 0°, were 1.56 and 1.33, respectively. The corresponding MREs were 4.16% and 5.60%, respectively. These results indicate that the joint exhibits relatively low resistance to tangential deformation under pneumatic actuation alone (Figure 7c,d).

After introducing the VSD and adopting the drive-based variable stiffness method, the tangential stiffness was significantly enhanced. Under forward bending, the tangential stiffness with unilateral VSD activation increased from 0.173 N/mm at 0° to 0.832 N/mm at 40°, corresponding to a stiffness ratio of 4.80. The corresponding MRE was 6.76% (Figure 7e). Under reverse bending, the tangential stiffness increased from 0.182 N/mm at 0° to 0.762 N/mm at 40°, corresponding to a stiffness ratio of 4.18. The corresponding MRE was 3.63% (Figure 7f).

At a bending angle of 40°, the forward tangential stiffness was 0.167 N/mm without VSD and 0.832 N/mm with unilateral VSD activation. Under reverse loading at the same bending angle, the corresponding tangential stiffness values were 0.163 N/mm without VSD and 0.762 N/mm with unilateral VSD activation. Thus, unilateral VSD activation increased the forward and reverse tangential stiffness to approximately 4.98 and 4.67 times the corresponding values without VSD, respectively. These quantitative experimental results clearly demonstrate that unilateral VSD activation substantially increases the tangential stiffness of the flexible joint, thereby effectively improving its resistance to deformation under external loads.

Comparison of the forward and reverse bending results shows that the overall tangential stiffness under forward bending is higher than that under reverse bending after VSD installation. This difference becomes more evident at larger bending angles. This phenomenon is attributed to the differences in particle confinement state and force chain distribution under different loading paths, resulting in an asymmetric stiffness enhancement effect, which is consistent with the theoretical analysis results. Post-test inspection revealed no leakage, particle wear, or structural degradation, indicating reliable operation of the VSD under the tested conditions.

The comparisons between the theoretical predictions and experimental results are shown in Figure 7e,f. Under both forward and reverse bending conditions, the theoretical curves agree well with the experimental results in terms of the overall variation trend, particularly the nonlinear increase in stiffness with increasing bending angle. To further quantify these changes, the tangential stiffness values at bending angles of 0° and 40°, together with the corresponding stiffness ratios under different bending directions and VSD conditions, are summarized in Table 3. These results demonstrate that the established model can effectively describe the tangential stiffness characteristics of the joint.

### 4.3. Discussion

The bending–stiffness coupling originates from the interaction between the particle-jamming state and joint deformation. When the inner air chamber of the VSD is pressurized, it first expands radially and squeezes the particle filler between the inner and outer air chambers and drives the particles and outer air chamber into the grooves of the gap constraint rings. As the VSD pressure increases, the particles are gradually compressed within the confined space and form frictional locking and particle jamming against the groove walls, thereby increasing the VSD stiffness. Meanwhile, joint bending changes the groove gaps and the confinement and loading states of the embedded particles, causing the particle-induced deformation resistance and resisting moments to vary with the bending angle. Therefore, the tangential stiffness is affected by both the VSD pressure and bending angle, resulting in bending–stiffness coupling.

The current prototype is relatively large and heavy, which may limit its integration into compact flexible robotic systems and its application in space-constrained environments. The present study primarily focused on the experimental validation of the prototype’s structural principle and mechanical performance, while its miniaturization and lightweight design still require further optimization. Future work will focus on reducing the dimensions of the variable stiffness device and constraint structures and adopting lightweight materials for non-load-bearing components while preserving the current operating-pressure range and stiffness-regulation performance as far as possible.

The driving-force correction coefficient $K_{p}$ was determined experimentally by comparing the measured output force of the self-developed artificial muscle with its theoretical driving force. The model was quantitatively validated using the averaged results of five repeated experiments. The MREs of the bending-angle model are 10.12% without VSD and 6.77% with VSD, while those of the tangential-stiffness model range from 3.63% to 6.76% under the four tested conditions. Although the theoretical predictions agree satisfactorily with the experimental results, $K_{p}$ was calibrated for the current prototype and may require recalibration when the joint geometry or operating conditions change. Future work will improve the generality of the model by reducing its dependence on empirical calibration.

The joint successfully operated with a 1 kg load, providing functional evidence of its load-handling capability under the tested condition. In the loaded-motion experiment, the joint angle continued to increase slowly after the initial transient response when the VSD was not activated. After VSD activation, the joint underwent a short transient oscillation and subsequently maintained a stable bending angle, indicating improved pose retention under the external load. The tangential stiffness experiment further provides quantitative evidence of the joint’s resistance to load-induced deformation. At a bending angle of 40°, unilateral VSD activation increased the forward and reverse tangential stiffness to approximately 4.98 and 4.67 times the corresponding values without VSD, respectively, indicating an improved resistance to deformation under external loading. However, the present experiments do not constitute a systematic maximum-load test, and the maximum supported load at different stiffness levels has not yet been quantified. Future work will conduct incremental loading and long-term holding tests under different VSD pressures and bending angles to further evaluate the maximum supported load and pose drift.

## 5. Conclusions

This paper focuses on the difficulty in balancing compliance and load-bearing capacity of actively variable stiffness pneumatic flexible bending joints. A structural scheme of a novel actively variable stiffness pneumatic flexible bending joint is proposed to realize joint stiffness adjustment under various working conditions. A theoretical model describing the relationship between bending angle and stiffness characteristics is established, and the effectiveness of the proposed method is validated through prototype experiments.

(1) A novel actively variable stiffness pneumatic flexible bending joint is developed. While maintaining flexibility and compliance, the joint exhibits enhanced stiffness and pose-retention capability.
(2) A theoretical model of the bending angle is established and experimentally verified. The model predictions agree well with the experimental results and capture the nonlinear positive correlation between the joint bending angle and air pressure. At 0.4 MPa, the measured forward bending angles of the flexible joint without and with VSD are 102.32° and 56.35°, respectively. The corresponding MREs are 10.12% and 6.77%, respectively. The reduction in bending angle is attributed to the inherent stiffness of the inactive VSD, which produces an antagonistic effect on joint bending. The bending joint equipped with VSD presents stable overall deformation and a regular motion trajectory.
(3) Without VSD, the joint exhibits relatively low resistance to deformation in the tangential loading direction. Unilateral VSD activation significantly enhances the tangential stiffness under both forward and reverse loading. Under forward loading, the tangential stiffness increases from 0.167 N/mm without VSD to 0.832 N/mm with VSD activation at a bending angle of 40°, while the corresponding values at 0° are 0.107 N/mm and 0.173 N/mm, respectively. Accordingly, the stiffness ratio $k_{t}(40^{\circ })/k_{t}(0^{\circ })$ increases from 1.56 to 4.80. Under reverse loading, VSD activation increases the tangential stiffness from 0.163 N/mm to 0.762 N/mm at 40° and from 0.123 N/mm to 0.182 N/mm at 0°, with the corresponding stiffness ratio increasing from 1.33 to 4.18. At a bending angle of 40°, the tangential stiffness with VSD activation is approximately 4.98 and 4.67 times that without VSD under forward and reverse loading, respectively. These results quantitatively demonstrate the stiffness-regulation capability of the proposed joint.

This study provides a design and modeling approach for actively variable stiffness flexible bending joints and supports their further development in flexible robotic systems. Future research will focus on dynamic modeling and experiments under complex loading conditions to extend the applicability of the joint to rehabilitation, service, and other robotic systems.

## Figures and Tables

**Table 1:** Key parameters of the actively variable stiffness pneumatic flexible bending joint.

Parameter	Value	Unit
Joint mass	1338.8	g
Diameter of Pneumatic artificial muscle constraint ring	Φ25	mm
Diameter of gap constraint ring	Φ35.8	mm
Clearance between constraint rings	1.6	mm
Thickness of constraint ring	3	mm
Distance from Pneumatic artificial muscle to neutral layer	16.89	mm
Distance from VSD to neutral layer	25.9	mm
Elastic steel plate dimensions	290.04 × 73.6 × 0.5	mm
Number of constraint rings	53	-
Pneumatic artificial muscle dimensions	Φ20 × 24	mm
Inner air chamber dimensions of VSD	Φ8 × 12	mm
Outer air chamber dimensions of VSD	Φ20 × 24	mm
Number of gap constraint rings	16	-

**Table 2:** Parameters used in the analytical model and their determination methods.

Parameter	Physical Meaning	Value	Unit	Determination Method
$l_{s}$	Effective length of the joint	290.04	mm	Measured from the prototype
$D_{1}$	Initial outer diameter of the artificial muscle	24	mm	Component specification
$D_{2}$	Initial inner diameter of the artificial muscle	20	mm	Component specification
$L_{x}$	Distance from the artificial-muscle axis to the centerline of the elastic steel plate	16.89	mm	Determined from the prototype geometry
b	Width of the elastic steel plate	73.6	mm	Measured from the prototype
t	Thickness of the elastic steel plate	0.5	mm	Component specification
E	Elastic modulus of the artificial-muscle material	1.042	MPa	Material test or manufacturer data
μ	Poisson’s ratio of the rubber material	0.3	–	Assumed based on material properties
$K_{p}$	Driving-force correction coefficient of the artificial muscle	1.52	–	Identified experimentally
P4	Operating pressure	0–0.4	MPa	Experimental operating range

**Table 3:** Tangential stiffness under different bending directions and VSD conditions.

Bending Direction	VSD Condition	k_t_ at 0° (N/mm)	k_t_ at 40° (N/mm)	k_t_(40°)/k_t_(0°)
Forward	Without VSD	0.107	0.167	1.56
Forward	Unilateral VSD activation	0.173	0.832	4.80
Reverse	Without VSD	0.123	0.163	1.33
Reverse	Unilateral VSD activation	0.182	0.762	4.18

## Data Availability

The raw data supporting the conclusions of this article will be made available by the authors on request.

## References

[1] Cesário L.C., Barbosa P., Miguel P.A.C., Mendes G.H.S. (2025). Service Robots in Caring for Older Adults: Uncovering the Current Conceptual and Intellectual Structures and Future Research Agenda. Arch. Gerontol. Geriatr..

[2] Lim W.M., Jasim K.M., Malathi A. (2025). Service Robots in Healthcare: Toward a Healthcare Service Robot Acceptance Model (sRAM). Technol. Soc..

[3] Balcha H.B., Rauffet P., Wegari G.M., Buche C. (2025). A Review on Human–Robot Trust in Home Service Robots. ACM Trans. Hum.-Robot Interact..

[4] Jin T., Han X. (2024). Robotic Arms in Precision Agriculture: A Comprehensive Review of the Technologies, Applications, Challenges, and Future Prospects. Comput. Electron. Agric..

[5] Pietrantoni L., Favilla M., Fraboni F., Mazzoni E., Morandini S., Benvenuti M., De Angelis M. (2024). Integrating Collaborative Robots in Manufacturing, Logistics, and Agriculture: Expert Perspectives on Technical, Safety, and Human Factors. Front. Robot. AI.

[6] Zhou S., Li Y., Wang Q., Lyu Z. (2024). Integrated Actuation and Sensing: Toward Intelligent Soft Robots. Cyborg Bionic Syst..

[7] Khan S.U.E., Varghese R.J., Kassanos P., Farina D., Burdet E. (2026). Space Physiology and Technology: Adaptations, Countermeasures, and Opportunities for Wearable Systems. Cyborg Bionic Syst..

[8] AboZaid Y.A., Aboelrayat M.T., Fahim I.S., Radwan A.G. (2024). Soft Robotic Grippers: A Review on Technologies, Materials, and Applications. Sens. Actuators A Phys..

[9] Seleem I.A., Naeem M.A., Takemura H., Ishii H. (2025). Tendon-Driven Based Extensible Soft Robot with Variable Stiffness. Robot. Auton. Syst..

[10] Wang K., Sun L., Liu Z., Sun N., Zhang J. (2025). Actuators and Variable Stiffness of Flexible Surgical Actuators: A Review. Sens. Actuators A Phys..

[11] Jiang P., Ma T., Luo J., Yang Y., Yin C., Zhong Y. (2025). A Reconfigurable Soft Helical Actuator with Variable Stiffness Skeleton. Soft Robot..

[12] Yang Y., Wang P., Zhu H., Xia K., Ren T., Shen Y., Li Y. (2024). A Variable Stiffness Soft Robotic Manipulator Based on Antagonistic Design of Supercoiled Polymer Artificial Muscles and Shape Memory Alloys. Sens. Actuators A Phys..

[13] Park W., Lee D., Bae J. (2022). A Hybrid Jamming Structure Combining Granules and a Chain Structure for Robotic Applications. Soft Robot..

[14] Zhang Z., Yu W., Zhao J., Li Y., Meng C., Guo S. (2026). A Review of Fiber-Shape Rolled Dielectric Elastomer Actuators: A Pivotal Pathway in Advancing Bionic Actuation. Small.

[15] Aghajani S., Wu C., Li Q., Fang J. (2024). Additively Manufactured Composite Lattices: A State-of-the-Art Review on Fabrications, Architectures, Constituent Materials, Mechanical Properties, and Future Directions. Thin-Walled Struct..

[16] Guo H., Xu X., Liu H., Zhang W., Wang X., Liu Z., Li X., Liu Y., Zhao G., Zhang Y. (2026). Innovative Soft Material-Assisted Robot Grasping Devices: From Design Concept to Fabrication and Application Scenarios. J. Field Robot..

[17] Cortes Lizarazo J.R., Miranda Pulido C.E. (2025). Discrete Element Method Simulation of Contact Evolution in Granular Jamming Grippers. MATTER Int. J. Sci. Technol..

[18] Song C., Lee H.S., Park S., Hwang D. (2025). Stiffening Iron Particles to Modulate Physical Interactions. Nat. Commun..

[19] Hrehova S., Hošovský A., Husár J., Krenický T. (2026). Granular Jamming in Soft Robotics: Simulation Frameworks and Emerging Possibilities—Review. Biomimetics.

[20] Amend J.R., Brown E., Rodenberg N., Jaeger H.M., Lipson H. (2012). A Positive Pressure Universal Gripper Based on the Jamming of Granular Material. IEEE Trans. Robot..

[21] Liu T., Xia H., Lee D.-Y., Firouzeh A., Park Y.-L., Cho K.-J. (2021). A Positive Pressure Jamming Based Variable Stiffness Structure and Its Application on Wearable Robots. IEEE Robot. Autom. Lett..

[22] Peng Y., Yuan J., Hu Z., Du L., Bao S., Magdy M. A Soft Robotic Gripper with Variable Grasping Force Based on Jamming Phenomenon. Proceedings of the 2025 IEEE International Conference on Mechatronics and Automation (ICMA).

[23] Wang S., Wang W. (2026). A Stiffness-Tunable Soft Actuator with Adjustable Jamming for Dexterous Manipulation. Soft Robot..

[24] Caro F., Carmichael M.G. (2024). A Review of Mechanisms to Vary the Stiffness of Laminar Jamming Structures and Their Applications in Robotics. Actuators.

[25] Chen T., Yang X., Zhang B., Li J., Pan J., Wang Y. (2024). Scale-Inspired Programmable Robotic Structures with Concurrent Shape Morphing and Stiffness Variation. Sci. Robot..

[26] Wang Z., Shi X., Zhang G., Wang W.D. (2026). An Origami-Based Anthropomorphic Rotary Robotic Arm. Int. J. Mech. Sci..

[27] Molla M.H.O.R., Chen J., Xu C. (2026). Advancing Soft Robotics: Recent Progress in Dielectric Elastomer and Fluid Actuators. npj Robot..

[28] Cortes J., Miranda C. (2025). Design, Control, and Applications of Granular Jamming Grippers in Soft Robotics. Robotics.

[29] Copaci D.-S., Blanco D., Martin-Clemente A., Moreno L. (2020). Flexible Shape Memory Alloy Actuators for Soft Robotics: Modelling and Control. Int. J. Adv. Robot. Syst..

[30] Pagliarani N., Arleo L., Albini S., Cianchetti M. (2023). Variable Stiffness Technologies for Soft Robotics: A Comparative Approach for the STIFF-FLOP Manipulator. Actuators.

[31] Bermeo J.E., Castillo-Castañeda E., Laribi M.A. (2025). A Soft Variable Stiffness Actuator with a Chain Mail Structure as a Particle Jamming Interface. Actuators.

[32] Enyan M., Bing Z., Amu-Darko J.N.O., Issaka E., Otoo S.L., Agyemang M.F. (2025). Advances in Smart Materials Soft Actuators on Mechanisms, Fabrication, Materials, and Multifaceted Applications: A Review. J. Thermoplast. Compos. Mater..

[33] Gent A.N., Zhang L., Tian M., Liu L., Feng Y. (2002). Engineering with Rubber: How to Design Rubber Components.

[34] Hibbeler R.C. (2021). Engineering Mechanics: Statics.

[35] Gere J.M., Goodno B.J. (2019). Mechanics of Materials.

